# The effect of lichen secondary metabolites on *Aspergillus* fungi

**DOI:** 10.1007/s00203-021-02649-0

**Published:** 2021-12-29

**Authors:** Łukasz Furmanek, Paweł Czarnota, Mark R. D. Seaward

**Affiliations:** 1 Department of Ecology and Environmental Protection, University of Rzeszów, ul. Zelwerowicza 4, 35-601 Rzeszów, Poland; 2 School of Archaeological and Forensic Sciences, University of Bradford, Bradford, BD7 1DP UK

**Keywords:** Lichen extracts, *Aspergillus*, Inhibition, Antifungal potential, Allelopathy

## Abstract

A systematic review of literature data on the antifungal potential of extracted lichen compounds and individual secondary metabolites against mold species of the genus *Aspergillus* is provided. Crude extracts from 49 epiphytic, 16 epigeic and 22 epilithic species of lichens and 44 secondary metabolites against 10 species, *Aspergillus candidus*, *A. flavus*, *A. fumigatus*, *A. nidulans*, *A. niger*, *A. ochraceus*, *A. parasiticus*, *A. restrictus*, *A. stellatus* and *A. ustus*, were analysed. Several measuring techniques were employed for such analyses. Lichen substances were extracted with alcoholic and other organic solvents mainly using the Soxhlet apparatus. Among the three most-studied mold species, the results showed that the crude extracts from the thalli of the lichens *Cladonia foliacea*, *Hypotrachyna cirrhata*, *Leucodermia leucomelos*, *Platismatia glauca* and *Pseudevernia furfuracea* against *Aspergillus flavus*, from *C. foliacea*, *Nephroma arcticum* and *Parmelia sulcata* against *A. fumigatus* and from *Evernia prunastri*, *Hypogymnia physodes*, *Umbilicaria cylindrica* and *Variospora dolomiticola* against *A. niger* have the greatest antifungal potential. The lichen secondary metabolites showed a higher inhibitory potential, e.g. protolichesterinic acid against *A. flavus*, lecanoric acid against *A. fumigatus* and orsellinic acid against *A. nige*r; the other seven species of *Aspergillus* have been poorly studied and require further investigation. A comparison of the inhibitory potential of the tested mixtures of lichen substances and their secondary metabolites shows that they can compete with commonly used antifungal substances, such as ketoconazole and clotrimazole against *A. flavus*, *A. nidulans*, *A. niger* and *A. parasiticus* and fluconazole in the case of *A. fumigatus*.

**Supplementary Information:**

The online version contains supplementary material available at 10.1007/s00203-021-02649-0.

## Introduction

Lichens are fundamentally a symbiotic system consisting of a photosynthetic alga or cyanobacterium and heterotrophic fungus, usually an Ascomycota (Armaleo et al. 2019), but occasionally a Basidiomycota (Furmanek et al. 2019) (172 species: 0.9%: Lücking et al. 2017); to date, there are c. 20,000 known lichen species (Lücking et al. 2017; Armaleo et al. 2019). Considerable interest has been shown in the metabolites biosynthesized in this symbiosis, which may depend on the relation of lichen bionts, as in the case of the presence of association-related bacteria (Calcott et al. 2018).

The lichen produces a wide range of primary metabolites, such as polysaccharides, proteins, dyes (chlorophyll, carotenoids) and vitamins (Elix and Stocker-Wörgötter 2008; Spribille et al. 2020), but due to the complexity of the symbiosis, it is not always possible to accurately determine the origin of their biosynthesis (Elix and Stocker-Wörgötter 2008; Ranković and Kosanić 2015). In general, primary metabolic substances dissolve in hot water (Elix and Stocker-Wörgötter 2008) and polysaccharides even in cold water (Surayot et al. 2019). The dominant lichen sugars include α- and β-glucan and galactomannan (Akbulut and Yildiz 2010; Boustie et al. 2011), the solubility of which depend on the bond ratio (1 → 3) to (1 → 4) α-d-glucan (Carbonero et al. 2001; Cordeiro et al. 2005); in a mixture of extracted compounds, they may constitute the dominant part (up to 57%) (Akbulut and Yildiz 2010; Boustie et al. 2011). The qualitative composition of polysaccharides depends on their synthesis by the biocomponents of lichens, mainly the mycobiont (Cordeiro et al. 2004; Akbulut and Yildiz 2010), but it may result from their metabolic pathway (Cordeiro et al. 2004) and is independent of the availability of nutrients (Cordeiro et al. 2011).

The research on the biological activity of lichen polysaccharides is poorly understood, although their immunomodulatory (Freysdottir et al. 2008; Surayot et al. 2019) and anticancer activities (Akbulut and Yildiz 2010) have been confirmed. The antifungal potential of polysaccharides (lichenan: Ruotsalainen et al. 2009), as for other lichen primary substances, awaits more detailed investigation.

An important role is played by secondary compounds biosynthesized by the fungal component in the sugar transformation process photosynthesized by the photobiont (Calcott et al. 2018; Furmanek 2019; Furmanek et al. 2019).

The dry mass weight of secondary metabolites in lichens ranges from 1 to 30% (Honegger 2009; Gadea et al. 2017; Furmanek et al. 2019). Their solubility in water is poor (Furmanek 2019), but lichen polyphenols (Zagoskina et al. 2013), which include a significant proportion of secondary metabolites (atranorin or usnic acid) (Fernández-Moriano et al. 2015; Prokopyev et al. 2018), are soluble in water, as well as in organic solvents (Komaty et al. 2016; Pereira et al. 2017; Furmanek 2019).

The effectiveness of rainwater as a solvent depends upon its level of pH, with low solubility occurring in acidic and neutral water (Honegger 2009) and a possible negative influence on the stability of the compound structure at a radically high or low pH (as for atranorin in alcoholic and other organic solvents: Vos et al. 2018). The solubility level is also increased by specific functional groups attached to the secondary compound, e.g. hydroxyl groups –OH (Rundel 1978). Currently, more than 1000 secondary metabolites are known (Goga et al. 2018; Furmanek et al. 2019), most of which are biosynthesized in the acetate–polymalonate pathway; these include, for example, compounds classified as anthraquinones, derivatives of usnic acid (dibenzofurans), higher aliphatic fatty acids or depsons and depsidones. The mevalonic acid pathway is represented by steroids, carotenoids and terpenoids (e.g. zeorin) (Elix 2014; Goga et al. 2018; Furmanek et al. 2019). Some of these are derived via the shikimic acid pathway, i.e. the class of pulvinic acid derivatives (Elix 2014; Goga et al. 2018; Furmanek et al. 2019). Most of the lichen secondary compounds are specific to lichenized fungi not occurring in any other living organisms (Molnár and Farkas 2010; Furmanek et al. 2019).

Contrary to primary metabolites, secondary compounds have a wide spectrum of activities, from antioxidant (Fernández-Moriano et al. 2015 and literature cited therein), anticancer (Solárova et al. 2020 and literature cited therein) to antifungal potentials, as in the case of *Aspergillus* molds reviewed in this study.

The species-rich genus of *Aspergillus*, apart from the *Penicillium* and *Fusarium* genera, forms a dominant part of the fungi. It gains an advantage due to its increased growth capacity under high temperature conditions and its lower humidity requirements and with a longer period of sporulation. Their spores withstand higher stress conditions in light and show resistance to several chemicals (Pitt and Hocking 2009).

According to Chróst (2016) the genus *Aspergillus* consists of over 350 species with a distinctive conidiophore vesicle in the form of a specifically different shape. Conidia-forming cells (phialides) are developing on conidiophores, with conidiospores produced in the apical part. Species are based on the shape of conidiospores.

Representatives of the genus *Aspergillus* (Ascomycota) are an important source of mycotoxin production (Bennett and Klich 2003). They can be the cause of cancer diseases (Ahmed Adam et al. 2017; Claeys et al. 2020 and literature cited in both works), such as the genetic mutation associated with the p53 protein suppressor gene (Kim et al. 2016; Mantovani et al. 2019). Furthermore, mold mycotoxins, which are secondary metabolites (Park et al. 2015; Frisvad et al. 2018) of these fungi, are a source of other health problems, such as those related to the respiratory system (Bennett and Klich 2003; Park et al. 2015) and to autism (Ratnaseelan et al. 2018), as well as contributing to the development of diseases related to the auditory system (Pathak et al. 2013; Frejo et al. 2018). For a detailed description of the impact of mold mycotoxins on human health, see, for example, Bbosa et al. (2013).

Mycotoxins belonging to the class of aflatoxins or ochratoxins (Bennett and Klich 2003) are of high importance to agriculture (Esmaeilishirazifard and Keshavarz 2014) and human health (Chróst 2016). The most dangerous species are *Aspergillus flavus*, *A. fumigatus* and *A. ochraceus* (Chróst 2016). The toxicity of mycotoxins occurs at a low concentration (Frisvad et al. 2018). The physical properties of aflatoxins include resistance by their chemical structure to high temperature, low solubility in water, but high in moderately polar solvents (i.e. chloroform) and fluorescence under UV radiation (blue light—aflatoxin B; green—aflatoxin G) (Kumar 2018). Mold mycotoxins have caused poisoning on a greater scale in the past (Peraica et al. 1999). In addition to the known methods of chemical decomposition of aflatoxins, such as high temperature, biological agents are also used, such as certain species of bacteria or fungi (Kumar 2018), whose growth substances, investigated in this study, may be a future biological agent against *Aspergillus* spp.

Increasing resistance of representatives of the genus *Aspergillus* to the triazole class of antibiotics (itraconazole, voriconazole, isavuconazole and posaconazole) (van der Linden et al. 2011; Meis et al. 2016) is a continuing problem. Optimal growth conditions for *Aspergillus* fungi occur in habitats or climates with high humidity and temperature (Peraica et al. 1999; Chróst 2016) and sanitary safety areas in public buildings (especially hospitals and health care centres), as well as private buildings. Some of the mold species, namely *A. flavus*, *A. fumigatus*, *A. niger*, *A. ochraceus*, *A. ustus* and *A. versicolor* investigated in this study, have been identified in hospitals (Gniadek et al. 2011). Buildings which do not meet the requirements for the prevention of mold growth are known to cause so-called sick-building syndrome (Bennett and Klich 2003; Chróst 2016).

The priority of this analysis is the medical point of view, i.e. the therapeutic use of lichen substances in the prevention and treatment of human health pathologies caused by the presence of *Aspergillus* spp. and the sanitary safety of buildings through the use of lichen compounds as potential antibiotics. Researchers may also use the results as a source of fungistatic activity against *Aspergillus* spp. as plant pathogens (Pawar et al. 2008; Martínez et al. 2017). However, it should not be forgotten that *Aspergillus* spp. infecting food plants during their growth (Leger et al. 2000; Paica et al. 2013; Sharma et al. 2014) or during their storage (Perrone et al. 2007; Amaike and Keller 2011; Sharma et al. 2014) can contaminate food and thus reduce food security, and in consequence, not only result in health risks, but also large economic losses (Sharma et al. 2014; Mitchell et al. 2016). Consumption of infected food by livestock and humans leads to side-effects and re-emphasizes the role of lichen-crude extracts, including individual secondary metabolites as potential therapeutic substances (in the context of antibiotic activity), plant disease control agents (fungicides) and as food preservatives.

The ultimate aim of this review is to show the protective potential of lichen extracts and their secondary compounds against fungi from the genus of *Aspergillus* and their usable application in the future. Although the literature data seems generally significant, it covers only a relatively small number of the species from this genus that have been subjected to experiments.

## Materials and methods

The basis of this study was a systematic review of literature data on the effects of lichen extracts from lichen thalli (Table S1) and individual lichen secondary metabolites (Table S2) on 10 *Aspergillus* species: *A. candidus* Link, *A. flavus* Link [= *A. oryzae* (Ahlb.) Cohn], *A. fumigatus* Fresen., *A. nidulans* (Eidam) G. Winter, *A. niger* Tiegh., *A. ochraceus* G. Wilh., *A. parasiticus* Speare [= *A. sojae* Sakag. & K. Yamada], *A. restrictus* G. Sm., *A. stellatus* Curzi [= *A. variecolor* (Berk. & Broome) Thom & Raper] and *A. ustus* (Bainier) Thom & Church*.* The literature on the subject has provided data on the impact of the lichen crude extracts from 87 lichen species, composed of 49 epiphytic, 16 epigeic and 22 epilithic species (Table S1). A division based on the substrate was applied for easier interpretation of tables and analysis of results. If a lichen species occurs on more than one substrate, such as soil (epigeic lichens) and bark of wooded plants (epiphytic lichens), the species was included in the potentially dominant ecological group.

The solvent or solvents used in the process of extracting the lichen substances from the thalli are provided in Tables 1, 2, 3, 4, 5, Tables S3, S4, S5, S6, S7; however, despite the large amount of data, it is not stated whether the extracted compounds were re-dissolved (after solvent evaporation) in another solvent, e.g., in dimethylsulfoxide (DMSO), before the mycelium was properly treated with a particular extract. Appropriate information for the most effective extracts as well as for the process of extraction are mentioned if necessary. Analogously, the classification of secondary metabolites (Tables 6, 7, 8, Tables S8, S9, S10) based on the division employed by Elix (2014), namely 44 secondary compounds, divided into 11 biochemical classes (Table S2), was followed.

**Table 1:** Effect of crude extracts from epiphytic lichens on *Aspergillus flavus*

Lichen species	Extracting solvent	Results				Measurement method	Literature
		MIC [mg·ml^−1^]	MFC [mg·ml^−1^]	IZ [mm] (dose)	Other		
*Alectoria sarmentosa*	Ethanol	> 100	> 100	11 (20 µl)	‡	BTDM; AWDM	[1]
	Ethyl acetate	> 200	†	†			
	Water	> 200	†	†			
*Anaptychia ciliaris*	Methanol	†	‡	†	‡	BMM; DDM	[2]
*Bulbothrix setschwanensis*	Acetone	‡	‡	c. 8 (1 ml)	‡	DDM	[3]
	Chloroform			c. 7 (1 ml)			
	Methanol			†			
*Dirinaria consimilis*	Methanol	‡	‡	‡	IR: 63.63% (0.5 mg of extract·ml^−1^ of medium)	PFT	[4]
*Evernia divaricata*	Methanol	‡	‡	†	‡	DDM	[5]
*Evernia prunastri*	Acetone	12.5	‡	‡	‡	BMM	[6]
	Methanol	‡	‡	†	‡	DDM	[5]
		1.25	5	‡	‡	MMwR	[7]
		‡	‡	†	‡	DDM	[8]
*Flavoparmelia caperata*	Acetone	†	‡	†	‡	BTDM; DDM	[9]
		6.25		9.3 (50 µl)			[10]
		50	‡	‡	‡	BMM	[11]
	Chloroform	6.25	‡	8.6 (50 µl)	‡	BTDM; DDM	[10]
	Ethanol	6.25	‡	16 (15 µl)	‡	BTDM; DDM	[9]
	Methanol	2.5	5	‡	‡	MMwR	[7]
		6.25	‡	8.6 (50 µl)	‡	BTDM; DDM	[10]
	Water	†	‡	†	‡	BTDM; DDM	[9]
*Heterodermia diademata*	Acetone	‡	‡	c. 11 (1 ml)	‡	DDM	[3]
	Chloroform			†			
	Methanol			c. 13 (1 ml)			
*Heterodermia obscurata*	Methanol	‡	‡	‡	IR: 36.36% (0.5 mg of extract·ml^−1^ of medium)	PFT	[4]
*Hypogymnia physodes*	Acetone	25	‡	‡	‡	BMM	[12], [13]
		12.5		14 (15 µl)		BTDM; DDM	[9]
	Ethanol	12.5	‡	16 (15 µl)	‡	BTDM; DDM	[9]
	Methanol	12.5	‡	‡	‡	BMM	[13]
	Water	†	‡	‡	‡	BMM	[13]
		†	‡	†	‡	BTDM; DDM	[9]
*Hypotrachyna cirrhata*	Water	‡	‡	‡	SGI: 100% (80 µl·ml^−1^)	SGI	[14]
*Hypotrachyna nepalensis*	Acetone	‡	‡	c. 14 (1 ml)	‡	DDM	[3]
	Chloroform			†			
	Methanol			c. 14 (1 ml)			
*Letharia vulpina*	Methanol	‡	‡	20 (10–200 µl)	‡	DDM	[8]
				†			
*Leucodermia leucomelos*	Water	‡	‡	‡	SGI: 100% (80 µl·ml^−1^)	SGI	[15]
*Menegazzia terebrata*	Acetone	12.5	‡	12 (15 µl)	‡	BTDM; DDM	[9]
		6.25		‡		BMM	[12]
	Ethanol	6.25		15 (15 µl)		BTDM; DDM	[9]
	Water	†		†			
*Ochrolechia androgyna*	Acetone	†	‡	†	‡	BTDM; DDM	[16]
	Methanol						
	Water						
*Parmelia omphalodes*	Acetone	6.25	‡	22 (15 µl)	‡	BTDM; DDM	[17]
	Methanol	6.25		20 (15 µl)			
	Water	†		†			
*Parmelia saxatilis*	Acetone	25	‡	‡	‡	BMM	[11]
				13 (15 µl)		BTDM; DDM	[18]
	Methanol	12.5	‡	‡	‡	BMM	[19]
		‡		†		DDM	[20]
		12.5		17 (15 µl)		BTDM; DDM	[18]
	Water	†	‡	†	‡	BTDM; DDM	[18]
*Parmelia sulcata*	Acetone	12.5	‡	‡	‡	BMM	[11]
		50		20 (15 µl)		BTDM; DDM	[21]
		6.25		23 (15 µl)			[18]
	Methanol	1.25	2.5	‡	‡	MMwR	[7]
		50	‡	18 (15 µl)		BTDM; DDM	[21] [18]
		3.12	‡	23 (15 µl)			
	Water	†	‡	†	‡	BTDM; DDM	
*Parmeliopsis ambigua*	Acetone	25	‡	‡	‡	BMM	[12]
				14 (15 µl)		BTDM; DDM	[18]
	Methanol	12.5	‡	‡	‡	BMM	[19]
				16 (15 µl)		BTDM; DDM	[18]
	Water	†	‡	†	‡	BTDM; DDM	[18]
*Parmeliopsis hyperopta*	Acetone	12.5	‡	13 (15 µl)	‡	BTDM; DDM	[22] [23]
	Methanol	6.25		16 (15 µl)			
	Water	†		†			
*Parmotrema andinum*	Acetone	‡	‡	7.33 (15 µl)	‡	DDM	[24]
	2-propanol			15.66 (15 µl)			
*Parmotrema crinitum*	Methanol	7.5	‡	12 (10 µl)	‡	BTDM; DDM	[25]
*Parmotrema grayanum*	Methanol	‡	‡	‡	IR: c. 38% (1 mg·ml^−1^ of medium)	PFT	[26]
*Parmotrema praesorediosum*	Methanol	‡	‡	‡	IR: 60% (1 mg·ml^−1^ of medium)		
*Parmotrema reticulatum*	Acetone	25	‡	14 (50 µl)	IR: 64%	BTDM; DDM	[27]
		12.5		13 (15 µl)	‡		[17]
	Chloroform	25	‡	9.3 (50 µl)	IR: 43%		[27]
	Methanol	25	‡	13.6 (50 µl)	IR: 62%		[27]
		12.5		15 (15 µl)	‡		[17]
		‡	‡	‡	IR: 27.27% (0.5 mg of extract·ml^−1^ of medium)	PFT	[4]
	Water	†	‡	†	‡	BTDM; DDM	[17]
*Parmotrema thomsonii*	Acetone	‡	‡	c. 15 (1 ml)	‡	DDM	[3]
	Chloroform			†			
	Methanol			†			
*Parmotrema tinctorum*	Acetone	‡	‡	12.6 (5 ml)	‡	DDM	[28]
	Chloroform	‡	‡	8 (5 ml)	‡		
	Methanol	‡	‡	11.3 (5 ml)	‡	DDM	[28]
				‡	IR: c. 42% (1 mg·ml^−1^ of medium)	PFT	[26]
	2-propanol	‡	‡	10 (15 µl)	‡	DDM	[29]
*Physcia aipolia*	Acetone	6.25	‡	12 (15 µl)	‡	BTDM; DDM	[16]
	Methanol	25		12 (15 µl)			
	Water	†		†			
*Platismatia glauca*	Acetone	> 2.5	> 2.5	‡	‡	MMwR	[30]
	Ethyl acetate	> 2.5	> 2.5	‡	‡		
	Methanol	0.63	0.63	‡	‡		
		‡	‡	†	‡	DDM	[20]
*Protousnea poeppigii*	Methanol	> 0.25	‡	‡	‡	BMM	[31]
	Methylene chloride						
*Pseudevernia furfuracea*	Acetone	0.31	> 2.5	‡	‡	MMwR	[30]
		12.5	‡			BMM	[6]
	Ethyl acetate	12.5	> 2.5	‡	‡	MMwR	[30]
	Methanol	‡	‡	†	‡	DDM	[8]
		0.04	0.63	‡		MMwR	[30]
		‡	‡	†	‡	DDM	[8]
*Ramalina conduplicans*	Ethanol	‡	‡	‡	IR: c. 42% (1 mg of extract·ml^−1^ of medium)	PFT	[32]
	Ethyl acetate				IR: c. 50% (1 mg of extract·ml^−1^ of medium)		
	Petroleum ether				IR: c. 35% (1 mg of extract·ml^−1^ of medium)		
*Ramalina hossei*	Methanol	‡	‡	‡	IR: 63.63% (0.5 mg of extract·ml^−1^ medium)	PFT	[4]
*Ramalina pacifica*	Methanol	‡	‡	‡	IR: 51.51% (0.5 mg of extract·ml^−1^ medium)		
*Ramalina pollinaria*	Methanol	‡	‡	†	‡	DDM	[20]
*Ramalina polymorpha*	Methanol	‡	‡	†	‡		
*Stereocaulon paschale*	Methanol	10	‡	‡	‡	BMM	[33]
*Teloschistes flavicans*	Acetone	‡	‡	10 (concent. 1000 mg·ml^−1^)	‡	DDM	[34]
*Usnea barbata*	Acetone	12.5	‡	‡	‡	BMM	[35]
*Usnea florida*	Methanol	> 0.25	‡	‡	‡	BMM	[31]
	Methylene chloride						
*Vulpicida pinastri*	Methanol	7.5	‡	20 (10 µl)	‡	BTDM; DDM	[25]

Method of measurement abbreviations: *BMM* Broth microdilution method (MIC), *BTDM* Broth tube dilution method (MIC), *DDM* Disk diffusion method (IZ), *PFT* Poisoned Food Technique (fungal diameter), *MMwR* Microdilution method with resazurin (MIC, MFC), *AWDM* Agar well diffusion method (IZ), *SGI* Spore Germination Inhibition (%), *MIC* Minimal Inhibitory Concentration, *MFC* Minimal Fungicidal Concentration, *IZ* Inhibition Zone. Literature abbreviations: [1] Ranković and Mišić (2007); [2] Ranković et al. (2010c); [3] Tiwari et al. (2011b); [4] Prashith Kekuda et al. (2015); [5] Aslan et al. (2006); [6] Kosanić et al. (2013a); [7] Mitrović et al. (2011); [8] Kirmizigül et al. (2003); [9] Ranković et al. (2009); [10] Babiah et al. (2014b); [11] Kosanić et al. (2012a); [12] Kosanić et al. (2013b); [13] Kosanić and Ranković (2011b); [14] Shahi et al. (2003); [15] Shahi et al. (2001); [16] Ranković et al. (2007b); [17] Kosanić and Ranković (2011a); [18] Ranković and Kosanić (2012); [19] Kosanić et al. (2014a); [20] Gulluce et al. (2006); [21] Ranković et al. (2007a); [22] Ranković et al. (2010b); [23] Kosanić et al. (2010); [24] Anjali et al. (2015a); [25] Ranković et al. (2010a); [26] Vivek et al. (2014); [27] Babiah et al. (2014a); [28] Tiwari et al. (2011a); [29] Anjali et al. (2015b); [30] Mitrović et al. (2014); [31] Schmeda-Hirschmann et al. (2008); [32] Yashoda et al. (2014); [33] Ranković et al. (2014a); [34] Maulidiyah et al. (2018); [35] Ranković et al. (2012).

^†^No effect, ^‡^not investigated, *IR* inhibition rate (%)

**Table 2:** Effect of crude extracts from epiphytic lichens on *Aspergillus fumigatus*

Lichen species	Extracting solvent	Results				Measurement method	Literature
		MIC [mg·ml^−1^]	MFC [mg·ml^−1^]	IZ [mm] (dose)	Other		
*Alectoria sarmentosa*	Ethanol	150	200	9 (20 µl)	‡	BTDM, AWDM	[1]
	Ethyl acetate	> 200	†	11 (20 µl)	‡		
	Water	> 200	†	†	‡		
*Bulbothrix setschwanensis*	Acetone	‡	‡	c. 12 (1 ml)	‡	DDM	[2]
	Chloroform	‡	‡	c. 10 (1 ml)	‡		
	Methanol	‡	‡	c. 5 (1 ml)	‡		
*Evernia prunastri*	Acetone	12.5	‡	‡	‡	BMM	[3]
	Methanol	1.25	1.25	‡	‡	MMwR	[4]
		‡	‡	†	‡	DDM	[5]
*Flavoparmelia caperata*	Acetone	25	‡	9 (15 µl)	‡	BTDM; DDM	[6]
		25	‡	‡	‡	BMM	[7]
	Ethanol	3.12	‡	15 (15 µl)	‡	BTDM; DDM	[6]
	Methanol	1.25	1.25	‡	‡	MMwR	[4]
	Water	†	‡	†	‡	BTDM; DDM	[6]
*Heterodermia diademata*	Acetone	‡	‡	c. 13 (1 ml)	‡	DDM	[2]
	Chloroform	‡	‡	†	‡		
	Methanol	‡	‡	c. 15 (1 ml)	‡		
*Hypogymnia physodes*	Acetone	12.5	‡	‡	‡	BMM	[8], [9]
		12.5	‡	15 (15 µl)	‡	BTDM; DDM	[6]
	Ethanol	6.25	‡	14 (15 µl)	‡	BTDM; DDM	[6]
	Methanol	1.25	1.25	‡	‡	MMwR	[4]
		6.25	‡	‡	‡	BMM	[9]
	Water	†	‡	†	‡	BTDM; DDM	[6]
*Hypotrachyna cirrhata*	Water	‡	‡	‡	SGI: 100% (concent. 80 µl·ml^−1^)	SGI	[10]
*Hypotrachyna nepalensis*	Acetone	‡	‡	c. 10 (1 ml)	‡	DDM	[2]
	Chloroform	‡	‡	†	‡		
	Methanol	‡	‡	c. 8 (1 ml)	‡		
*Letharia vulpina*	Methanol	‡	‡	†	‡	DDM	[5]
*Leucodermia leucomelos*	Water	‡	‡	‡	SGI: 100% (concent. 80 µl·ml^−1^)	SGI	[11]
*Menegazzia terebrata*	Acetone	6.25	‡	14 (15 µl)	‡	BTDM; DDM	[6]
		6.25	‡	‡	‡	BMM	[8]
	Ethanol	3.12	‡	17 (15 µl)	‡	BTDM; DDM	[6]
	Water	†	‡	†	‡		
*Ochrolechia androgyna*	Acetone	†	‡	†	‡	BTDM; DDM	[12]
	Methanol						
	Water						
*Parmelia omphalodes*	Acetone	6.25	‡	16 (15 µl)	‡	BTDM; DDM	[13]
	Methanol	6.25	‡	18 (15 µl)	‡		
	Water	†	‡	†	‡		
*Parmelia saxatilis*	Acetone	25	‡	‡	‡	BMM	[7]
		25	‡	18 (15 µl)	‡	BTDM; DDM	[14]
	Methanol	6.25	‡	24 (15 µl)	‡		
	Water	†	‡	†	‡		
*Parmelia sulcata*	Acetone	1280 µg (concent 3 mg·ml^−1^)	‡	‡	‡	DDM	[15]
		6.25	‡	‡	‡	BMM	[7]
		25	‡	14 (15 µl)	‡	BTDM; DDM	[16]
		6.25	‡	18 (15 µl)	‡		[14]
	Chloroform	120 µg (concent. 1.12 mg·ml^−1^)	‡	‡	‡	DDM	[15]
	Diethyl ether	105 µg (concent. 0.5 mg·ml^−1^)	‡	‡	‡		
	Methanol	320 µg (concent. 6 mg·ml^−1^)	‡	‡	‡		
		< 9.8 × 10^–3^	< 9.8 × 10^–3^	‡	‡	MMwR	[4]
		25	‡	18 (15 µl)	‡	BTDM; DDM	[16]
		3.12	‡	19 (15 µl)	‡		[14]
	Water	†	‡	†	‡	BTDM; DDM	[16], [14]
*Parmeliopsis ambigua*	Acetone	25	‡	†	‡	BMM	[8]
		25	‡	14 (15 µl)	‡	BTDM; DDM	[14]
	Methanol	6.25	‡	16 (15 µl)	‡	BTDM; DDM	[14]
	water	†	‡	†	‡		
*Parmeliopsis hyperopta*	Acetone	12.5	‡	14 (15 µl)	‡	BTDM; DDM	[17], [18]
	Methanol	3.12	‡	17 (15 µl)	‡		
	Water	†	‡	†	‡		
*Parmotrema perlatum*	Hexane	1.25	‡	14 (5 µl)	‡	BMM; DDM	[19]
*Parmotrema reticulatum*	Acetone	12.5	‡	15 (15 µl)	‡	BTDM; DDM	[13]
	Methanol	6.25	‡	21 (15 µl)	‡		
	Water	†	‡	†	‡		
*Parmotrema thomsonii*	Acetone	‡	‡	c. 12 (1 ml)	‡	DDM	[2]
	Chloroform	‡	‡	c. 6 (1 ml)	‡		
	Methanol	‡	‡	c. 7 (1 ml)	‡		
*Parmotrema tinctorum*	Acetone	‡	‡	12 (5 ml)	‡	DDM	[20]
	Chloroform	‡	‡	19.6 (5 ml)	‡		
	Methanol	‡	‡	18.7 (5 ml)	‡		
*Physcia aipolia*	Acetone	6.25	‡	12 (15 µl)	‡	BTDM; DDM	[12]
	Methanol	12.5	‡	11 (15 µl)	‡		
	Water	†	‡	†	‡		
*Protousnea poeppigii*	Methanol	> 0.25	‡	‡	‡	BMM	[21]
	Methylene chloride						
*Pseudevernia furfuracea*	Acetone	12.5	‡	‡	‡	BMM	[3]
	Methanol	‡	‡	†	‡	DDM	[5]
*Ramalina hossei*	Acetone	‡	‡	12 (concent. 20 mg·ml^−1^)	‡	AWDM	[22]
	Chloroform	‡	‡	11 (concent. 20 mg·ml^−1^)	‡		
	Ethyl acetate	‡	‡	9 (concent. 20 mg·ml^−1^)	‡		
	Methanol	‡	‡	14 (concent. 20 mg·ml^−1^)	‡		
	Petroleum ether	‡	‡	9 (concent. 20 mg·ml^−1^)	‡		
*Usnea barbata*	Acetone	12.5	‡	‡	‡	BMM	[23]
*Usnea florida*	Methanol	> 0.25	‡	‡	‡	BMM	[21]
	Methylene chloride						

Method of measurement abbreviations: *BMM* Broth microdilution method (MIC), *BTDM* Broth tube dilution method (MIC), *DDM* Disk diffusion method (IZ)/(MIC), *MMwR* Microdilution method with resazurin (MIC, MFC), *AWDM* Agar well diffusion method (IZ), *SGI* Spore Germination Inhibition (%). For MIC, MFC and IZ abbreviations: see Table 1. Literature abbreviations: [1] Ranković and Mišić (2007); [2] Tiwari et al. (2011b); [3] Kosanić et al. (2013a); [4] Mitrović et al. (2011); [5] Kirmizigül et al. (2003); [6] Ranković et al. (2009); [7] Kosanić et al. (2012a); [8] Kosanić et al. (2013b); [9] Kosanić and Ranković (2011b); [10] Shahi et al. (2003); [11] Shahi et al. (2001); [12] Ranković et al. (2007b); [13] Kosanić and Ranković (2011a); [14] Ranković and Kosanić (2012); [15] Candan et al. (2007); [16] Ranković et al. (2007a); [17] Ranković et al. (2010b); [18] Kosanić et al. (2010); [19] Hoda and Vijayaraghavan (2015); [20] Tiwari et al. (2011a); [21] Schmeda-Hirschmann et al. (2008); [22] Praveen Kumar et al. (2010); [23] Ranković et al. (2012)

^†^No effect, ^‡^not investigated

**Table 3:** Effect of crude extracts from epigeic lichens on *Aspergillus fumigatus*

Lichen species	Extracting solvent	Results				Measurement method	Literature
		MIC [mg·ml^−1^]	MFC [mg·ml^−1^]	IZ [mm] (dose)	Other		
*Cladonia foliacea*	Methanol	3.13 × 10^–1^	2.5	‡	‡	MMwR	[1]
*Cladonia furcata*	Acetone	12.5	‡	12 (15 µl)	‡	BTDM; DDM	[2]
		12.5	‡	‡	‡	BMM	[3]
							[4]
							[5]
	Ethanol	12.5	‡	15 (15 µl)	‡	BTDM; DDM	[2]
	Methanol	12.5	‡	‡	‡	BMM	[5]
	Water	†	‡	†	‡	BTDM; DDM	[2]
		†	‡	‡	‡	BMM	[5]
*Cladonia pyxidata*	Acetone	25	‡	‡	‡	BMM	[4]
*Cladonia rangiferina*	Acetone	12.5	‡	‡	‡	BMM	[4]
	Ethanol	> 200	†	10 (20 µl)	‡	BTDM; AWDM	[6]
	Ethyl acetate	> 200	†	10 (20 µl)	‡		
	Water	> 200	†	†	‡		
*Nephroma arcticum*	Water	‡	‡	‡	Clear zone (fungicidal effect) (dose: 25 µl)	Cup plate method	[7]
		‡	‡	‡	Inhibition (dose: 3 drops × 25 µl)	Drop plate method	
*Peltigera aphthosa*	Water	‡	‡	‡	†	Cup plate method	[7]
		‡	‡	‡	†	Drop plate method	

Method of measurement abbreviations: *BMM* Broth microdilution method (MIC), *BTDM* Broth tube dilution method (MIC); *DDM* Disk diffusion method (IZ), *MMwR* Microdilution method with resazurin (MIC, MFC), *AWDM* Agar well diffusion method (IZ). For MIC, MFC and IZ abbreviations: see Table 1. Literature abbreviations: [1] Mitrović et al. (2011); [2] Ranković et al. (2009); [3] Ranković et al. (2011); [4] Kosanić et al. (2014b); [5] Kosanić and Ranković (2011b); [6] Ranković and Mišić (2007); [7] Land and Lundström (1998).

^†^No effect, ^‡^not investigated

**Table 4:** Effect of crude extracts from epiphytic lichens on *Aspergillus niger*

Lichen species	Extracting solvent	Results				Measurement method	Literature
		MIC [mg·ml^−1^]	MFC [mg·ml^−1^]	IZ [mm] (dose)	Other		
*Alectoria sarmentosa*	Ethanol	50	50	14 (20 µl)	‡	BTDM; AWDM	[1]
	Ethyl acetate	100	150	10 (20 µl)	‡		
	Water	> 200	†	†	‡		
*Anaptychia ciliaris*	Methanol	†	‡	†	‡	BTDM; DDM	[2]
*Evernia divaricata*	methanol	‡	‡	†	‡	DDM	[3]
*Evernia prunastri*	Methanol	‡	‡	20.3 (1 mg)	‡	DDM	[4]
		62.5 × 10^–3^	‡	31 (300 µg)	‡	BMM; DDM	[3]
		1.25	5	‡	‡	MMwR	[5]
		1.56 × 10^–1^	1.56 × 10^–1^	‡	‡		
*Flavoparmelia caperata*	Acetone	3.12	‡	12.6 (50 µl)	‡	BTDM; DDM	[6]
	Chloroform	3.12	‡	6.6 (50 µl)	‡	BTDM; DDM	[6]
	Methanol	‡	‡	22.3 (40 µl)	‡	DDM	[4]
		2.5	20	‡	‡	MMwR	[5]
		10	40	‡	‡		
		3.12	‡	12 (50 µl)	‡	BTDM; DDM	[6]
*Hypogymnia physodes*	Methanol	‡	‡	19.7 (40 µl)	‡	DDM	[4]
		5	10	‡	‡	MMwR	[5]
		< 9.8 × 10^–3^	< 9.8 × 10^–3^	‡	‡		
*Parmelia saxatilis*	Methanol	‡	‡	†	‡	DDM	[7]
*Parmelia sulcata*	Acetone	640 µg (concent 3 mg·ml^−1^)	‡	‡	‡	DDM	[8]
	Chloroform	240 µg (concent. 1.12 mg·ml^−1^)	‡	‡	‡		
	Diethyl ether	105 µg (concent. 0.5 mg·ml^−1^)	‡	‡	‡		
	Methanol	320 µg (concent. 6 mg·ml^−1^)	‡	‡	‡		
		‡	‡	16.3 (40 µl)	‡	DDM	[4]
		2.5	2.5	‡	‡	MMwR	[5]
		10	20	‡	‡		
*Parmotrema andinum*	Acetone	‡	‡	13 (15 µl)	‡	DDM	[9]
	2-propanol	‡	‡	21.33 (15 µl)	‡		
*Parmotrema crinitum*	Methanol	†	‡	†	‡	BTDM; DDM	[10]
*Parmotrema perlatum*	Hexane	2.5	‡	10.1 (5 µl)	‡	BMM; DDM	[11]
*Parmotrema reticulatum*	Acetone	6.25	‡	15.6 (50 µl)	IR: 65%	BTDM; DDM	[12]
	Chloroform	6.25	‡	8.3 (50 µl)	IR: 35%		
	Methanol	6.25	‡	12.6 (50 µl)	IR: 52%		
*Parmotrema tinctorum*	Acetone	‡	‡	14.7 (5 ml)	‡	DDM	[13]
	Chloroform	‡	‡	†	‡		
	Methanol	‡	‡	19 (5 ml)	‡		
	2-propanol	‡	‡	8.66 (15 µl)	‡	DDM	[14]
*Platismatia glauca*	Methanol	‡	‡	†	‡	DDM	[7]
*Protousnea poeppigii*	Methanol	> 0.25	‡	‡	‡	BMM	[15]
	Methylene chloride						
*Pseudevernia furfuracea*	Acetone	†	‡	‡	‡	DDM	[16]
	Chloroform	1813 µg (concent. 8.5 mg·ml^−1^)	‡	‡	‡	DDM	[16]
	Ethanol	‡	‡	‡	IS: 36.66% (375 µl of 10% concent. of extract)	IS	[17]
		800 µg (concent. 15 mg·ml^−1^)	‡	‡	‡	DDM	[16]
*Pseudevernia furfuracea* var. *ceratea*	Acetone	2390 µg (concent. 5.6 mg·ml^−1^)	‡	‡	‡	DDM	[16]
	Chloroform	3148 µg (concent. 7.4 mg·ml^−1^)	‡	‡	‡		
	Ethanol	2920 µg (concent. 6.8 mg·ml^−1^)	‡	‡	‡		
*Ramalina farinacea*	Chloroform	‡	‡	16 (4 mg)	‡	DDM	[18]
	Ethanol	‡	‡	22 (4 mg)	‡		
	N-hexane	‡	‡	20 (4 mg)	‡		
	Water	‡	‡	†	‡		
*Ramalina hossei*	Acetone	‡	‡	13 (concent. 20 mg·ml^−1^)	‡	AWDM	[19]
	Chloroform	‡	‡	10 (concent. 20 mg·ml^−1^)	‡		
	Ethyl acetate	‡	‡	11 (concent. 20 mg·ml^−1^)	‡		
	Methanol	‡	‡	13 (concent. 20 mg·ml^−1^)	‡		
	Petroleum ether	‡	‡	9 (concent. 20 mg·ml^−1^)	‡		
*Ramalina pollinaria*	Methanol	‡	‡	†	‡	DDM	[7]
*Ramalina polymorpha*	Methanol	‡	‡	†	‡	DDM	[7]
*Usnea antiqua*	Acetone	‡	‡	23 (0.1 ml)	‡	Cup plate agar diffusion method (IZ)	[20]
*Usnea barbata*	Acetone	‡	‡	‡	IR: 61.57% (concent. 10 mg·ml^−1^)	PFT	[21]
	Methanol	‡	‡	‡	IR: 36.97% (concent. 10 mg·ml^−1^)		
	Water	‡	‡	‡	IR: 0.63% (concent. 10 mg·ml^−1^)		
*Usnea complanata*	Acetone	‡	‡	13 (0.1 ml)	‡	Cup plate agar diffusion method (IZ)	[20]
*Usnea florida*	Methanol	> 0.25	‡	‡	‡	BMM	[15]
	Methylene chloride						
*Usnea maculata*	Acetone	‡	‡	18 (0.1 ml)	‡	Cup plate agar diffusion method (IZ)	[20]
*Usnea sanguinea*	Acetone	‡	‡	17 (0.1 ml)	‡		
*Usnea submollis*	Acetone	‡	‡	26 (0.1 ml)	‡		
*Vulpicida pinastri*	Methanol	3.75	‡	18 (10 µl)	‡	BTDM; DDM	[10]

Method of measurement abbreviations: *BMM* Broth microdilution method (MIC), *BTDM* Broth tube dilution method (MIC), *DDM* Disk diffusion method (IZ)/(MIC); *PFT* Poisoned Food Technique (fungal diameter); *MMwR* Microdilution method with resazurin (MIC, MFC), *AWDM* Agar well diffusion method (IZ), *IS* Inbihition of sporulation (%). For MIC, MFC and IZ abbreviations: see Table 1. Literature abbreviations: [1] Ranković and Mišić (2007); [2] Ranković et al. (2010c); [3] Aslan et al. (2006); [4] Stojanović et al. (2013); [5] Mitrović et al. (2011); [6] Babiah et al. (2014b); [7] Gulluce et al. (2006); [8] Candan et al. (2007); [9] Anjali et al. (2015a); [10] Ranković et al. (2010a); [11] Hoda and Vijayaraghavan (2015); [12] Babiah et al. (2014a); [13] Tiwari et al. (2011a); [14] Anjali et al. (2015b); [15] Schmeda-Hirschmann et al. (2008); [16] Türk et al. (2006); [17] Karabulut and Ozturk (2015); [18] Esimone and Adikwu (1999); [19] Praveen Kumar et al. (2010); [20] Mohammed (2013); [21] Madamombe and Afolayan (2003)

^†^No effect, ^‡^not investigated

**Table 5:** Effect of crude extracts from epiphytic and epigeic lichens on *Aspergillus restrictus*

Lichen habitat	Lichen species	Extraction solvent	Results			Measurement method	Literature
			MIC [mg·ml^−1^]	MFC [mg·ml^−1^]	IZ		
Epiphytic lichens	*Evernia prunastri*	Methanol	1.25	1.25	‡	MMwR	[1]
	*Flavoparmelia caperata*	Methanol	5	5	‡		
	*Hypogymnia physodes*	Methanol	1.25	1.25	‡		
	*Parmelia sulcata*	Methanol	6.25 × 10^–1^	6.25 × 10^–1^	‡		
Epigeic lichen	*Cladonia foliacea*	Methanol	2.5	10	‡		

Method of measurement abbreviations: MMwR = Microdilution method with resazurin (MIC, MFC). For MIC, MFC and IZ abbreviations: see Table 1. Literature abbreviations: [1] Mitrović et al. (2011)

‡ not investigated

**Table 6:** Effect of lichen secondary metabolites on *Aspergillus flavus*

Biochemical class	Secondary metabolites	Extracting solvents	Results				Measurement method	Literature
			MIC [mg·ml^−1^]	MFC	IZ [mm]	Other		
Aliphatic acid	Protolichesterinic acid	Acetone and methanol	8 × 10^–3^	‡	21 (concent. 8 µg·ml^−1^)	‡	BMM; DDM	[1]
Benzyl ester	Barbatolic acid	Methanol, chloroform and acetone	0.1	‡	‡	‡	MMwR	[2]
Monocyclic aromatic derivatives	Methyl orsellinate	Dichloromethane followed by methanol	‡	‡	‡	IR: 80% (concent. 200 µg·ml^−1^)	PFT	[3]
	Methyl β-orsellinate (methyl β-orcinolcarboxylate)		‡	‡	‡	IR: 90% (concent. 200 µg·ml^−1^)		
	Orsellinic acid	Ethanol–water–hydrogen chloride	9.8 × 10^–3^	‡	‡	‡	BMM	[4], [5]
Orcinol depsides	Divaricatic acid	Acetone, methanol and water	12.5	‡	‡	‡	BTDM	[6], [7]
		Dichloromethane and acetone or acetone (Piovano et al. 2000, 2001)	0.25	‡	‡	‡	Agar dilution method (MIC)	[8]
	Isodivaricatic acid (depside: Schmeda-Hirschmann et al. 2008)	Dichloromethane	> 0.25	‡	‡	‡	BMM	[9]
	Evernic acid	Acetone	1	‡	‡	‡	BMM	[10]
	Erythrin	Dichloromethane followed by methanol	‡	‡	‡	†	PFT	[3]
	Lecanoric acid	Acetone	1	‡	‡	‡	BMM	[11]
		Ethanol–water–hydrogen chloride	12.6 × 10^–3^	‡	‡	‡	BMM	[4], [5]
		Dichloromethane followed by methanol	‡	‡	‡	IR: 40% (concent. 200 µg·ml^−1^)	PFT	[3]
		Methanol and acetone	0.125	‡	‡	‡	BTDM	[12]
	Sekikaic acid	Dichloromethane followed by methanol	‡	‡	‡	†	PFT	[3]
	Sphaerophorin	Dichloromethane and acetone or acetone (Piovano et al. 2000, 2001)	0.25	‡	‡	‡	Agar dilution method (MIC)	[8]
	2'-O-methyl anziaic acid	Acetone	1	‡	‡	‡	BMM	[11]
β-orcinol depsides	Atranorin	Acetone	1	‡	‡	‡	BMM	[13], [14]
		Acetone and methanol	0.5	‡	‡	‡	BTDM	[15]
		Chloroform	32 × 10^–3^	‡	18 (concent. 32 µg·ml^−1^)	‡	BMM; DDM	[16]
		Dichloromethane and acetone or acetone (Piovano et al. 2000, 2001)	> 0.25	‡	‡	‡	Agar dilution method (MIC)	[8]
		Dichloromethane followed by methanol	‡	‡	‡	†	PFT	[3]
	Diffractaic acid	Dichloromethane and acetone or acetone (Piovano et al. 2000, 2001)	0.25	‡	‡	‡	Agar dilution method (MIC)	[8]
		Ethanol–water–hydrogen chloride	8.3 × 10^–3^	‡	‡	‡	BMM	[4], [5]
	Thamnolic acid	Methanol, chloroform and acetone	†	‡	‡	‡	BMM	[17]
	2-hydroxy-4-methoxy-3,6-dimethylbenzoic acid (depside: Aravind et al. 2014)	Chloroform	16 × 10^–3^	‡	23 (concent. 16 µg·ml^−1^)	‡	BMM; DDM	[16]
Orcinol depsidones	Α-collatolic acid	Dichloromethane and acetone or acetone (Piovano et al. 2000, 2001)	> 0.25	‡	‡	‡	Agar dilution method (MIC)	[8]
	Lobaric acid	Dichloromethane and acetone or acetone (Piovano et al. 2000, 2001)	> 0.25	‡	‡	‡	Agar dilution method (MIC)	[8]
		Dichloromethane followed by methanol	‡	‡	‡	IR: 90% (concent. 200 µg·ml^−1^)	PFT	[3]
	Physodic acid	Acetone	1	‡	‡	‡	BMM	[13], [10]
		Acetone and methanol	1	‡	‡	‡	BTDM	[15]
		Acetone, methanol and water	1	‡	‡	‡	BMM	[18]
	Variolaric acid	Dichloromethane and acetone or acetone (Piovano et al. 2000, 2001)	> 0.25	‡	‡	‡	Agar dilution method (MIC)	[8]
β-orcinol depsidones	Fumarprotocetraric acid	Acetone	0.25	‡	‡	‡	BMM	[14]
		Acetone, methanol and water	0.25	‡	‡	‡	BMM	[18]
		Dichloromethane and acetone or acetone (Piovano et al. 2000, 2001)	> 0.25	‡	‡	‡	Agar dilution method (MIC)	[8]
		Methanol and acetone	0.25	‡	‡	‡	BTDM	[12]
	Norstictic acid	Acetone	0.5	‡	‡	‡	BMM	[19]
		Ethanol–water–hydrogen chloride	13.8 × 10^–3^	‡	‡	‡	BMM	[4], [5]
	Pannarin	Dichloromethane and acetone or acetone (Piovano Et Al. 2000, 2001)	> 0.25	‡	‡	‡	Agar dilution method (MIC)	[8]
	1’-chloropannarin (depsidone: Huneck and lamb 1975)							
	Protocetraric acid	Acetone	1	‡	‡	‡	BMM	[20]
		Ethanol–water–hydrogen chloride	14.1 × 10^–3^	‡	‡	‡	BMM	[4], [5]
		Methanol and acetone	1	‡	‡	‡	BTDM	[12]
	Psoromic acid	Dichloromethane and acetone or acetone (Piovano et al. 2000, 2001)	> 0.25	‡	‡	‡	Agar dilution method (MIC)	[8]
	Salazinic acid	Acetone	1	‡	‡	‡	BMM	[20]
		Dichloromethane and acetone or acetone (Piovano et al. 2000, 2001)	> 0.25	‡	‡	‡	Agar dilution method (MIC)	[8]
	Stictic acid	Dichloromethane and acetone or acetone (Piovano et al. 2000, 2001)	> 0.25	‡	‡	‡	Agar dilution method (MIC)	[8]
		Methanol and acetone	1	‡	‡	‡	BTDM	[12]
	Vicanicin	Dichloromethane and acetone or acetone (Piovano Et Al. 2000, 2001)	> 0.25	‡	‡	‡	Agar dilution method (MIC)	[8]
Orcinol tridepside	Gyrophoric acid	Acetone	1.25	‡	‡	‡	BMM	[21]
		Acetone and methanol	0.5	‡	‡	‡	BTDM	[15]
		Acetone, methanol and water	1	‡	‡	‡	BMM	[18]
		Dichloromethane and acetone or acetone (Piovano et al. 2000, 2001)	> 0.25	‡	‡	‡	Agar dilution method (MIC)	[8]
Terpenoid	Zeorin (Hopane-6α,22-diol)	Acetone, methanol and water	6.25	‡	‡	‡	BTDM	[6], [7]
Usnic acid derivatives	Usnic acid	Acetone	0.5	‡	‡	‡	BMM	[13], [19], [20]
		Acetone and methanol	0.5	‡	‡	‡	BTDM	[15]
		Dichloromethane	> 0.25	‡	‡	‡	BMM	[9]
		Ethanol–water–hydrogen chloride	20.1 × 10^–3^	‡	‡	‡	BMM	[4], [5]
	( +)-Usnic acid	Chloroform	32 × 10^–3^	‡	17 (concent. 32 µg·ml^−1^)	‡	BMM; DDM	[16]

Method of measurement abbreviations: *BMM* = Broth microdilution method (MIC), *BTDM* Broth tube dilution method (MIC), *DDM* Disk diffusion method (IZ), *PFT* Poisoned Food Technique (fungal diameter), *MMwR* Microdilution method with resazurin (MIC, MFC). For MIC, MFC and IZ abbreviations: see Table 1. Literature abbreviations: [1] Sasidharan et al. (2014); [2] Sarıözlü et al. (2016); [3] Thadhani et al. (2012); [4] Hanuš et al. (2007); [5] Hanuš et al. (2008); [6] Ranković et al. (2010b); [7] Kosanić et al. (2010); [8] Piovano et al. (2002); [9] Schmeda-Hirschmann et al. (2008); [10] Kosanić et al. (2013a); [11] Ristić et al. (2016); [12] Ranković and Mišić (2008); [13] Ranković et al. (2014b); [14] Kosanić et al. (2014b); [15] Ranković et al. (2008); [16] Aravind et al. (2014); [17] Cankılıç et al. (2017); [18] Kosanić and Ranković (2011b); [19] Ranković et al. (2012); [20] Manojlović et al. (2012b); [21] Kosanić et al. (2014c). Biochemical classes are given according to Elix (2014) unless otherwise indicated

^†^No effect, ^‡^not investigated, *IR* inhibition rate

**Table 7:** Effect of lichen secondary metabolites on *Aspergillus fumigatus*

Biochemical class	Secondary metabolites	Extracting solvents	Results				Measurement method	Literature
			MIC [mg·ml^−1^]	MFC	IZ	Other		
Benzyl ester	Barbatolic acid	Methanol, chloroform and acetone	‡	‡	‡	IS	MMwR	[1]
Monocyclic aromatic derivative	Methyl haematommate	Methanol	‡	‡	† (disk applied before germination)	‡	DDM	[2]
			‡	‡	† (disk applied after germination)	‡		
Orcinol depsides	Evernic acid	Acetone	1	‡	‡	‡	BMM	[3]
	Divaricatic acid	Acetone, methanol and water	12.5	‡	‡	‡	BTDM	[4], [5]
	Isodivaricatic acid (depside: Schmeda-Hirschmann et al. 2008)	Dichloromethane	> 0.25	‡	‡	‡	BMM	[6]
	Lecanoric acid	Methanol and acetone	0.125	‡	‡	‡	BTDM	[7]
β-orcinol depsides	Atranorin	Acetone	0.5	‡	‡	‡	BMM	[8], [9]
		Acetone and methanol	0.25	‡	‡	‡	BTDM	[10]
	Thamnolic acid	Methanol, chloroform and acetone	0.4	‡	‡	‡	BMM	[11]
Orcinol depsidone	Physodic acid	Acetone	0.5	‡	‡	‡	BMM	[8], [3]
		Acetone and methanol	1	‡	‡	‡	BTDM	[10]
		Acetone, methanol and water	1	‡	‡	‡	BMM	[12]
β-orcinol depsidones	Fumarprotocetraric acid	Acetone	0.25	‡	‡	‡	BMM	[9]
		Acetone, methanol and water	0.25	‡	‡	‡	BMM	[12]
		Methanol and acetone	0.25	‡	‡	‡	BTDM	[7]
	Norstictic acid	Acetone	0.5	‡	‡	‡	BMM	[13]
	Protocetraric acid	Acetone	0.25	‡	‡	‡	BMM	[14]
		Methanol and acetone	0.5	‡	‡	‡	BTDM	[7]
	Salazinic acid	Acetone	1	‡	‡	‡	BMM	[14]
		Acetone, chloroform, diethyl ether and methanol	250 µg·41.7 µl^−1^	‡	‡	‡	DDM	[15]
	Stictic acid	Methanol and acetone	1	‡	‡	‡	BTDM	[7]
Orcinol tridepside	Gyrophoric acid	Acetone	0.625	‡	‡	‡	BMM	[16]
		Acetone and methanol	0.25	‡	‡	‡	BTDM	[10]
		Acetone, methanol and water	0.25	‡	‡	‡	BMM	[12]
Terpenoid	Zeorin (hopane-6α,22-diol)	Acetone, methanol and water	6.25	‡	‡	‡	BTDM	[4], [5]
Usnic acid derivative	Usnic acid	Acetone	0.25	‡	‡	‡	BMM	[8], [13], [14]
		Acetone and methanol	0.5	‡	‡	‡	BTDM	[10]
		Dichloromethane	> 0.25	‡	‡	‡	BMM	[6]

Method of measurement abbreviations: *BMM* Broth microdilution method (MIC), *BTDM* Broth tube dilution method (MIC), *DDM* Disk diffusion method (IZ)/(MIC), *MMwR* Microdilution method with resazurin (MIC, MFC). For MIC, MFC and IZ abbreviations: see Table 1. Literature abbreviations: [1] Sarıözlü et al. (2016); [2] Hickey et al. (1990); [3] Kosanić et al. (2013a); [4] Ranković et al. (2010b); [5] Kosanić et al. (2010); [6] Schmeda-Hirschmann et al. (2008); [7] Ranković and Mišić (2008); [8] Ranković et al. (2014b); [9] Kosanić et al. (2014b); [10] Ranković et al. (2008); [11] Cankılıç et al. (2017); [12] Kosanić and Ranković (2011b); [13] Ranković et al. (2012); [14] Manojlović et al. (2012b); [15] Candan et al. (2007); [16] Kosanić et al. (2014c). Biochemical classes are given according to Elix (2014) unless otherwise indicated

^†^No effect, ^‡^not investigated, *IS* inhibition of sporulation

**Table 8:** Effect of lichen secondary metabolites on *Aspergillus niger*

Biochemical class	Secondary metabolites	Extracting solvents	Results				Measurement method	Literature
			MIC [mg·ml^−1^]	MFC	IZ	Other (dose)		
Anthraquinones	Emodin	Ethanol	20 × 10^–3^	‡	‡	‡	BTDM	[1]
		Benzene	‡	‡	‡	IR: 25% (100 µg)	DDM	[2]
	Erythroglaucin	Benzene	‡	‡	‡	IR: 13% (100 µg)	DDM	[2]
	Fallacinal	Ethanol	40 × 10^–3^	‡	‡	‡	BTDM	[1]
		Benzene	‡	‡	‡	IR: 38% (100 µg)	DDM	[2]
	Fallacinol (teloschistin)	Ethanol	20 × 10^–3^	‡	‡	‡	BTDM	[1]
		Benzene	‡	‡	‡	IR: 20% (100 µg)	DDM	[2]
	Parietin (physcion)	Benzene	‡	‡	‡	IR: 20% (100 µg)	DDM	[2]
		Ethanol	40 × 10^–3^	‡	‡	‡	BTDM	[1]
		Methanol	‡	‡	‡	IR: 20%	DDM	[3]
	Parietinic acid	Ethanol	40 × 10^–3^	‡	‡	‡	BTDM	[1]
	Xanthorin	Benzene	‡	‡	‡	IR: 28% (100 µg)	DDM	[2]
benzyl ester	Barbatolic acid	Methanol, chloroform and acetone	†	‡	‡	‡	MMwR	[4]
MONOCYCLIC aromatic derivatives	Ethyl orsellinate	Chloroform	330–500 × 10^–3^	‡	‡	‡	BTDM	[5]
	Methyl haematommate	Methanol	‡	‡	† (disk applied before germination)	‡	DDM	[6]
			‡	‡	† (disk applied after germination)	‡		
	Methyl orsellinate	Chloroform	330–500 × 10^–3^	‡	‡	‡	BTDM	[5]
	Methyl β-orsellinate (methyl-β-orcinol carboxylate)	Chloroform	80–160 × 10^–3^	‡	‡	‡	BTDM	[5]
	Orsellinic acid	Ethanol–water–hydrogen chloride	12.6 × 10^–3^	‡	‡	‡	BMM	[7], [8]
Orcinol depsides	Divaricatic acid	Dichloromethane and acetone or acetone (Piovano et al. 2000, 2001)	> 250 × 10^–3^	‡	‡	‡	Agar dilution method (MIC)	[9]
	Isodivaricatic acid (depside: Schmeda-Hirschmann et al. 2008)	Dichloromethane	> 250 × 10^–3^	‡	‡	‡	BMM	[10]
	Lecanoric acid	Acetone	1	‡	‡	‡	BMM	[11]
		Ethanol–water–hydrogen chloride	3.9 × 10^–3^	‡	‡	‡	BMM	[7], [8]
	Olivetoric acid	Acetone	†	‡	‡	‡	DDM	[12]
	Sphaerophorin	Dichloromethane and acetone or acetone (Piovano et al. 2000, 2001)	> 250 × 10^–3^	‡	‡	‡	Agar dilution method (MIC)	[9]
β-orcinol depsides	Atranorin	Dichloromethane and acetone or acetone (Piovano et al. 2000, 2001)	> 250 × 10^–3^	‡	‡	‡	Agar dilution method (MIC)	[9]
	Chloroatranorin	Acetone	†	‡	‡	‡	DDM	[12]
	Diffractaic acid	Dichloromethane and acetone or acetone (Piovano et al. 2000, 2001)	> 250 × 10^–3^	‡	‡	‡	Agar dilution method (MIC)	[9]
		Ethanol–water–hydrogen chloride	7.4 × 10^–3^	‡	‡	‡	BMM	[7], [8]
	Thamnolic acid	Methanol, chloroform and acetone	†	‡	‡	‡	BMM	[13]
Orcinol depsidones	α-collatolic acid	Dichloromethane and acetone or acetone (Piovano et al. 2000, 2001)	> 250 × 10^–3^	‡	‡	‡	Agar dilution method (MIC)	[9]
	Llobaric acid							
	Variolaric acid							
	2'-O-methyl anziaic acid	Acetone	1	‡	‡	‡	BMM	[11]
β-orcinol depsidones	Fumarprotocetraric acid	Dichloromethane and acetone or acetone (Piovano et al. 2000, 2001)	> 250 × 10^–3^	‡	‡	‡	Agar dilution method (MIC)	[9]
	Norstictic acid	Ethanol–water–hydrogen chloride	8.6 × 10^–3^	‡	‡	‡	BMM	[7], [8]
	Pannarin	Dichloromethane and acetone or acetone (Piovano et al. 2000, 2001)	> 250 × 10^–3^	‡	‡	‡	Agar dilution method (MIC)	[9]
	1’-chloropannarin (depsidone: Huneck and Lamb 1975)							
	Protocetraric acid	Ethanol–water–hydrogen chloride	6.9 × 10^–3^	‡	‡	‡	BMM	[7], [8]
	Psoromic acid	Dichloromethane and acetone or acetone (Piovano et al. 2000, 2001)	> 250 × 10^–3^	‡	‡	‡	Agar dilution method (MIC)	[9]
	Salazinic acid	Acetone, chloroform, diethyl ether and methanol	250 µg·41.7 µl^−1^	‡	‡	‡	DDM	[14]
		Dichloromethane and acetone or acetone (Piovano et al. 2000, 2001)	> 250 × 10^–3^	‡	‡	‡	Agar dilution method (MIC)	[9]
	Stictic acid	Dichloromethane and acetone or acetone (Piovano et al. 2000, 2001)	> 250 × 10^–3^	‡	‡	‡	Agar dilution method (MIC)	[9]
	Vicanicin							
Orcinol tridepside	Gyrophoric acid	Dichloromethane and acetone or acetone (Piovano et al. 2000, 2001)	> 250 × 10^–3^	‡	‡	‡	Agar dilution method (MIC)	[9]
Usnic acid derivative	Usnic acid	Ethanol–water–hydrogen chloride	5.8 × 10^–3^	‡	‡	‡	BMM	[7], [8]
		Dichloromethane	> 250 × 10^–3^	‡	‡	‡	BMM	[10]

Method of measurement abbreviations: *BMM* Broth microdilution method (MIC), *BTDM* Broth tube dilution method (MIC), *MMwR* Microdilution method with resazurin, *DDM *Disk diffusion method (IZ)/(MIC)/(IR). For MIC, MFC and IZ abbreviations: see Table 1. Literature abbreviations: [1] Manojlović et al. (2002); [2] Manojlović et al. (2000); [3] Manojlović et al. (2005); [4] Sarıözlü et al. (2016); [5] Ingólfsdóttir et al. (1985); [6] Hickey et al. (1990); [7] Hanuš et al. (2007); [8] Hanuš et al. (2008); [9] Piovano et al. (2002); [10] Schmeda-Hirschmann et al. (2008); [11] Ristić et al. (2016); [12] Türk et al. (2006); [13] Cankılıç et al. (2017); [14] Candan et al. (2007). Biochemical classes are given according to Elix (2014) unless otherwise indicated

^†^No effect, ^‡^not investigated, *IR* inhibition rate

The main measurement technique was minimal inhibitory concentration (MIC) or, more rarely, minimal fungicidal concentration (MFC). As a complement to the MIC/MFC or the main method of measured inhibitory effects, researchers have employed techniques based on the measurement of the diameter (in millimeters) of the fungus inhibition zone (IZ) in culture. To obtain the results in the form of MIC/MFC, researchers have often used such research methods as broth microdilution method (BMM) (see method description: e.g. Schmeda-Hirschmann et al. 2008; Ranković et al. 2012), broth tube dilution method (BTDM) (see method description: e.g. Kosanić and Ranković 2011a; Babiah et al. 2014a) or microdilution method with resazurin (MMwR) (see method description: Mitrović et al. 2011, 2014). In the case of the IZ, the disk diffusion method (DDM) (see method description: e.g. Kosanić and Ranković 2011a; Babiah et al. 2014a) or the agar well diffusion method (AWDM) (see method description: Ranković and Mišić 2007; Praveen Kumar et al. 2010) were used, by which the diameter of the mycelial inhibition zone was most often measured and as well as the MIC. If there were some discrepancies between the above-mentioned test methods and the description in the original text, then it was included in one of the above-mentioned methods, such as Kirby and Bauer disk diffusion method (see method description: Babiah et al. 2014a).

One of the rare test methods found in this review is the poisoned food technique (PFT) (see method description: e.g. Vivek et al. 2014; Prashith Kekuda et al. 2015), which measures mycelium growth after specific growth times/intervals and is most conveniently expressed as a percentage of mycelium growth. Alternative methods used included spore germination inhibition (SGI) (see method description: Shahi et al. 2001, 2003) and inhibition of sporulation (IS) (see method description: Karabulut and Ozturk 2015), which expressed the percentage value of *Aspergillus* species spores germinated. In this paper the names of the original research methods that are distinguished by the adopted methodology (or it was not certain which main method they should be assigned to), i.e. the Agar Dilution Method (see method description: Piovano et al. 2002) by which the MIC was measured and the Cup Plate Agar Diffusion Method (IZ) (see method description: Mohammed 2013) or the Cup Plate Method and Drop Plate Method for which only the overall effect was reported (see method descriptions: Land and Lundström 1998). In most cases, the methods used by the researchers, based on the measurement of MIC or IZ, allow for a large comparison of the obtained results of the effect of extracted lichen compounds on *Aspergillus* spp., regardless of the differences resulting from the methodological combinations used.

The detailed description of the main research methods (BTDM, BMM, DDM) is presented by the Clinical and Laboratory Standards Institute (CLSI 2015a, 2017; b).

The main concentration unit for the test substances was given as mg ml^−1^; in some cases, these were converted to standardize the presented results. It was not always possible to give the diameter of the paper disk in DDM and substance concentration in the extracts used; if necessary, this information is mentioned in the text.

The most frequently used solvents in the extraction process were organic solvents such as methyl alcohol or acetone, but water was frequently used. Less common solvents include ethyl alcohol, 2-propanol, ethyl acetate, chloroform, diethyl ether, petroleum ether, methylene chloride, hexane, dichloromethane, hydrogen chloride and benzene. In some cases, a mixture of solvents was used.

In order to determine the level of the inhibitory potential of the fungal mycelium of the tested extracted lichen compounds as extracts and individual secondary metabolites, a comparison was made of the effectiveness of the strongest of them compared with the used antifungal reference substances against *Aspergillus* spp. (Table S12A, B). From this point of view, the greatest attention was paid to the description of combinations that could effectively compete with the potential of the antibiotics used.

The current names of lichen and fungi species are given according to *Index Fungorum*, with older synonyms used in original literature sources provided in square brackets [] (access: 17.11.2020).

Part of tables (Tables S1–12) were added as a supplementary material on the Archives of Microbiology official website.

## Results

### Analyses of crude extracts of various lichen thalli

#### Potential of extracted lichen secondary metabolites against *Aspergillus candidus*

The only lichen tested was the epiphytic species *Hypotrachyna cirrhata*, the substances extracted with water completely inhibiting the germination of *Aspergillus candidus* spores (SGI: 100% for the concentration at 80 µl ml^−1^) (Shahi et al. 2003).

#### Potential of extracted lichen secondary metabolites against *Aspergillus flavus*

##### Assays for epiphytic lichens

Crude extracts from 42 species of epiphytic lichens were tested against *Aspergillus flavus* (Table 1). The MIC ranged from 0.04 to 50 mg ml^−1^. Inhibition for concentrations < 1 mg ml^−1^ was obtained by methanol and acetone from *Pseudevernia furfuracea*, by methanol from *Platismatia glauca*, and by methanol and methylene chloride extracts from *Protousnea poeppigi* and *Usnea florida*. In the concentration range ≥ 1–5 mg ml^−1^ MIC, effective compounds were extracted from *Evernia prunastri*, *Flavoparmelia caperata*, *Parmelia sulcata* and *Platismatia glauca*. The inhibitory effectiveness of most of the extracts was at the higher MIC concentrations, which in the range of ≥ 5–10 mg ml^−1^ included extracts from *Parmotrema crinitum*, *Flavoparmelia caperata*, *Menegazzia terebrata*, *Parmelia omphalodes*, *P. sulcata*, *Parmeliopsis hyperopta*, *Physcia aipolia*, *Vulpicida pinastri* and *Stereocaulon paschale*. The effects of other extracts were found for MIC ≥ 10 mg ml^−1^, while all water extracted compounds proved to be ineffective. The high potential of the extracts (MIC ≤ 5 mg ml^−1^) was confirmed according to the MFC, the concentration of which did not exceed 5 mg ml^−1^ (Table 1).

Diameters of ≥ 20 mm IZ were achieved by alcohol extracted compounds from *Letharia vulpina*, *Parmelia omphalodes*, *P. sulcata* and *Vulpicida pinastri*, but no effects were found for all water-extracted compounds and some combinations using organic solvents (Table 1).

Water-extracted substances of *Hypotrachyna cirrhata* and *Leucodermia leucomelos* fully inhibited the germination of fungal spores (SGI: 80 µl ml^−1^), while combinations using the percentage of fungal inhibition were found to be effective at levels c. 28–64% (Table 1).

Extracted compounds from *Anaptychia ciliaris* (MIC, IZ), *Evernia divaricata* (IZ), *Ochrolechia androgyna* (MIC, IZ), *Ramalina pollinaria* and *R. polymorpha* (IZ) proved to be completely ineffective (Table 1).

##### Assays for epigeic lichens

Crude extracts from 10 species of epigeic lichens were tested against *Aspergillus flavus* (Table S3). The only remarkable MIC effectiveness was shown by methanol-extracted substances from *Cladonia foliacea* at a concentration of 15.62 × 10^–3^ mg ml^−1^, although the IZ did not confirm a strong inhibition. In a study by Mitrović et al. (2011), the same combination showed a weaker but distinct MIC value (2.5 mg ml^−1^), confirmed by the biocidal concentration (10 mg ml^−1^ MFC). Relatively stronger extracts proved to be compounds extracted with methanol from *Cetraria islandica* (5 mg ml^−1^ MIC). The MIC range of the other extracts was 12.5 → 200 mg ml^−1^. Water-based methodological combinations were ineffective (Table S3). The IZ values do not indicate a significant inhibition degree of the tested combinations, reaching a maximum of 13 mm in diameter (Ranković et al. 2009).

Substances extracted by water from *Cladonia furcata* (MIC, IZ) and by methanol from *C. fimbriata* and *C. digitata* (MIC, IZ) and by three solvents (IZ) from *C. rangiformis* were ineffective. (Table S3).

##### Assays for epilithic lichens

Crude extracts from 20 species of epilithic lichens were investigated against *Aspergillus flavus* (Table S4). Among these, the ethanol- and methanol-extracted substances from *Umbilicaria polyphylla* (1.56 mg ml^−1^) achieved the highest MIC value. The compounds extracted with methanol from *Physcia caesia* (3.12 mg ml^−1^) and acetone from *Xanthoparmelia xanthomelanea* (5 mg ml^−1^) proved to be less effective. The other data of epilithic lichen extracts do not provide distinct data against *Aspergillus flavus*, being active in the range of 6.25–50 mg ml^−1^. Some of the tested combinations were ineffective, including the thallus extracted with water (Table S4).

The highest IZ value (20 mm) confirmed the greater fungistatic effect of substances extracted with ethanol from *Umbilicaria polyphylla* and methanol from *Physcia caesia*. For other data, the IZ value ranges from 10 to 18 mm (Table S4).

Attempts made to extract compounds from *Arctoparmelia centrifuga*, *Aspicilia cinerea*, *Dermatocarpon miniatum*, *Lathagrium cristatum*, *Lecanora frustulosa*, *Ochrolechia parella*, *O. tartarea*, *Umbilicaria cylindrica*, *U. nylanderiana* and *Xanthoparmelia pulla* proved ineffective (Table S4).

#### Potential of extracted lichen secondary metabolites against *Aspergillus fumigatus*

##### Assays for epiphytic lichens

Crude extracts from 27 species of epiphytic lichens were investigated against *Aspergillus fumigatus* (Table 2), its sensitivity to these extracts ranging from < 9.8 × 10^–3^ to > 200 mg ml^−1^, which proved to be similar to those of *A. flavus*. For MFC value (< 9.8 × 10^–3^ mg ml^−1^), the methanol-extracted compounds from *Parmelia sulcata* were the most effective. However, other researchers have not confirmed the high effectiveness of this combination at concentrations of 3.12 mg ml^−1^ and 25 mg ml^−1^ MIC. The potential of *P. sulcata* substances in a methanol extract was 320 µg and was weaker than extracted compounds in diethyl ether (105 µg) and chloroform (120 µg), but stronger compared to the acetone extract (1280 µg). The potential of mixture substances extracted from *Protousnea poeppigii* and *Usnea florida*, by methanol and methylene chloride (> 0.25 mg ml^−1^) should also be highlighted. Compounds from *Evernia prunastri*, *Flavoparmelia caperata* and *Hypogymnia physodes* dissolved in methanol showed an inhibitory and fungicidal effect at the same concentration (1.25 mg ml^−1^), although the study of Kosanić and Ranković (2011b) for *H. physodes* shows a five times weaker value. The high potential was also unconfirmed by acetone-, ethanol- and water-extracted substances from *H. physodes*. The same inhibition potential (1.25 mg ml^−1^ MIC) was achieved by hexane—compounds from *Parmotrema perlatum* compared to ethanol**—**substances from *F. caperata*, *Menegazzia terebrata* and methanol-extracted substances from *Parmeliopsis hyperopta* (3.12 mg ml^−1^). The other combinations achieved lower MIC values, or they were not confirmed, as for all water extracts (Table 2).

The MIC value for the most effective combinations is not supported by the IZ values. The IZ value ≥ 20 mm is only confirmed by the methanol-extracted substances from *Parmelia saxatilis* (24 mm) and *Parmotrema reticulatum* (21 mm) at MIC of 6.25 mg ml^−1^. Below the diameter of 20 mm, IZ was a combination based on methanol- and chloroform-extracted compounds from *Parmotrema tinctorum*, but no MIC determination experiments were conducted. Substances extracted by methanol from *Parmelia omphalodes* by acetone (18 mm *vs* 6.25 mg ml^−1^), by methanol (19 mm *vs* 3.12 mg ml^−1^) from *P. sulcata*, and by ethanol from *Menegazzia terebrata*, and as well as by methanol from *Parmeliopsis hyperopta* (17 mm*vs* 3.12 mg ml^−1^), were slightly weaker according to the IZ diameter (Table 2).

*Aspergillus fumigatus* spores are completely susceptible to the presence of water-extracted substances (SGI for a concentration at 80 µl ml^−1^) from *Hypotrachyna cirrhata* and *Leucodermia leucomelos*. No antifungal potential was found for the extracted compounds from *Ochrolechia androgyna* and *Letharia vulpina* (Table 2).

##### Assays for epigeic lichens

Crude extracts from six species of epigeic lichens were investigated against *Aspergillus fumigatus* (Table 3). The effectiveness of substances extracted with methanol from *Cladonia foliacea* for its biocidal potential was 2.5 mg ml^−1^ MFC and fungistatic potential was 3.13 × 10^–1^ mg ml^−1^ MIC. *Nephroma arcticum* water-extracted compounds also had a biocidal effect. The effectiveness of other tested combinations ranged from 12.5 mg ml^−1^ to > 200 mg ml^−1^ MIC. Water-based combinations from *Cladonia furcata*, *C. rangiferina* and *Peltigera aphthosa* proved to be ineffective. The inhibition zone of the four combinations was in the range of 10–15 mm (Table 3).

##### Assays for epilithic lichens

Crude extracts from 13 species of epilithic lichens were tested against *Aspergillus fumigatus* (Table S5). Among the range of inhibition potential of this ecological group of lichens against *A. fumigatus*, two combinations using *Umbilicaria polyphylla* based on ethanol- and methanol-extracted substances (1.56 mg ml^−1^ MIC) are of the greatest importance, with a high IZ value (30 mm). The acetone combination from *Xanthoparmelia xanthomelanea* (2.5 mg ml^−1^) and methanol-extracted substances from *Tephromela atra* (3.12 mg ml^−1^ MIC *vs* 15 mm IZ), as well as from *Physcia caesia* (3.12 mg ml^−1^ MIC *vs* 23 mm IZ) were weaker. The inhibition potentials of the other substance mixtures were in the range of 6.25–25 mg ml^−1^ or proved to be ineffective (Table S5).

From an IZ point of view, in addition to those mentioned, the diameter of 20 mm was achieved by the mixtures of substances extracted with acetone from *Physcia caesia*, and by methanol from *Lasallia pustulata* (6.25 mg ml^−1^ MIC *vs* 19 mm IZ) and *Umbilicaria crustulosa* (6.25 mg ml^−1^ MIC *vs* 18 mm IZ) (Table S5). All experimentally extracted compounds from *Aspicilia cinerea*, *Lathagrium cristatum* and *Umbilicaria cylindrica* were unsuccessful (Table S5).

#### Potential of extracted lichen secondary metabolites against *Aspergillus niger*

##### Assays for epiphytic lichens

Crude extracts from 29 species of epiphytic lichens were tested against *Aspergillus niger* (Table 4). The antifungal potential of epiphytic lichen species against *A. niger* corresponds to the range of effectiveness for *A. flavus* and *A. fumigatus*.

The most effective MIC value is < 9.8 × 10^–3^ mg ml^−1^ and corresponds to the substances dissolved with methanol from *Hypogymnia physodes*; however, other data provided in the same study by Mitrović et al. (2011) show a much broader and less effective possibility of influencing the fungus species studied, also in the context of fungicidal effects (MFC). The second highest MIC value concerns the crude extract by methanol from *Evernia prunastri*, the concentration of which was 62.5 × 10^–3^ mg ml^−1^ in one study, which proved to be sufficient and confirmed by a high inhibition zone (31 mm); however, Mitrović et al. (2011) did not find an equally high MIC, but a positive MFC result highlights the efficacy of the extracted substances from *E. prunastri*. Potentially, highly effective crude extracts (methanol, methylene chloride) originated from *Protousnea poeppigii* and *Usnea florida* (> 0.25 mg ml^−1^ MIC). In addition, all methanol combinations with proven fungicidal effectiveness (MFC) showed their potential usefulness, and even weaker potential of substances from *Alectoria sarmentosa*. *Parmotrema perlatum* thalli subjected to hexane extraction indicates on an inhibition effect by the isolated substances (2.5 mg ml^−1^), but the IZ value (10.1 mm) did not confirm this. The other crude extracts were within the range of 3.12–6.25 mg ml^−1^ or had no effect. Some data indicated that combinations from *Parmelia sulcata* (105–640 µg) were most effective when compared with *Pseudevernia furfuracea* (800–1813 µg) and *P. furfuracea* var. *ceratea* (2390–3148 µg) (Table 4).

Several IZ values, in addition to the previously mentioned IZ for the *Evernia prunastri* combination, showed a higher antifungal potential. These include extracted compounds from *Usnea submollis* (26 mm), *U. antiqua* (23 mm), *Parmotrema andinum* (21.33 mm), *Ramalina farinacea* (20 and 22 mm) and *Flavoparmelia caperata* (22.3 mm). However, since the MIC was not investigated, these combinations become less relevant.

Compounds extracted by acetone, methanol and water from *Usnea barbata* reduced the relative mycelial growth by c. 1–62% when compared with the higher effectiveness substances extracted by acetone, methanol and chloroform from *Parmotrema reticulatum* (35–65%) and reduced sporulation (c. 37%) with the use of ethanol-extracted compounds from *Pseudevernia furfuracea* (Table 4).

The inhibitory potentials of extracted substances from *Anaptychia ciliaris*, *Evernia divaricata*, *Parmelia saxatilis*, *Parmotrema crinitum*, *Platismatia glauca*, *Ramalina pollinaria* and *R. polymorpha* were not confirmed (Table 4).

##### Assays for epigeic lichens

Crude extracts from nine species of epigeic lichens were tested against *Aspergillus niger* (Table S6). The epigeic lichen data were not characterized by a high inhibitory efficiency, except for species of *Peltigera*, whose substances were extracted with water and methanol (5 × 10^–3^ mg ml^−1^). Combinations with fungicidal activity are worth emphasizing, such as methanol-extracted compounds from *Cladonia foliacea*, as well as much weaker ethanol- and ethyl acetate-extracted substances from *C. rangiferina*. *Aspergillus niger* turned out to be resistant to an extract obtained from *Gyalolechia fulgens* and an unnamed *Cladonia* species (Table S6).

##### Assays for epilithic lichens

Crude extracts from 10 species of epilithic lichens against *Aspergillus niger* (Table S7) were tested, among which combinations using *Umbilicaria cylindrica* were undeniably outstanding. The substances present in the methanol extract were more strongly inhibited (15.62 × 10^–3^ mg ml^−1^ MIC), while the effectiveness of compounds in chloroform extracts was two times weaker. The inhibition potential of ethanol-extracted substances from *Variospora dolomiticola* (160 × 10^–3^ mg ml^−1^) is also apparent. There was no inhibition effect for compounds extracted from *Dermatocarpon miniatum*, *Nephroma parile*, *Ochrolechia parella*, *Umbilicaria nylanderiana* and *Xanthoparmelia pulla* (Table S7).

#### Potential of extracted lichen secondary metabolites against *Aspergillus parasiticus*

The substances extracted with water from two epiphytic lichens, *Hypotrachyna cirrhata* (Shahi et al. 2003) and *Leucodermia leucomelos* (Shahi et al. 2001), completely inhibited the germination of *A. parasiticus* spores (SGI: 100% for a concentration at 80 µl ml^−1^).

#### Potential of extracted lichen secondary metabolites against *Aspergillus restrictus*

Against *Aspergillus restrictus*, crude extracts from four epiphytic lichen species (*Evernia prunastri*, *Flavoparmelia caperata*, *Hypogymnia physodes* and *Parmelia sulcata*) and one epigeic species (*Cladonia foliacea*) were investigated. The MIC values ranged from 6.25 × 10^–1^ to 5 mg ml^−1^, and apart from the extracted substances from *C. foliacea* (2.5 mg ml^−1^ MIC; 10 mg ml^−1^ MFC), obtained identical MFC results (Mitrović et al. 2011) (Table 5).

#### Potential of extracted lichen secondary metabolites against *Aspergillus stellatus*

Against *Aspergillus stellatus*, crude extracts from six epiphytic lichen species (*Evernia divaricata*, *E. prunastri*, *Parmelia saxatilis*, *Platismatia glauca*, *Ramalina pollinaria* and *R. polymorpha*), two epigeic species (*Cladonia foliacea* and *C. rangiformis*) and three epilithic species (*Dermatocarpon miniatum*, *Umbilicaria nylanderiana* and *Xanthoparmelia pulla*) were investigated. None of the combinations tested showed antifungal effects according to the Disk diffusion method (IZ). *Evernia divaricata*, *E. prunastri*, *Cladonia foliacea*, *Dermatocarpon miniatum* and *Xanthoparmelia pulla* (Aslan et al. 2006) and *Parmelia saxatilis*, *Platismatia glauca*, *Ramalina pollinaria*, *R. polymorpha* and *Umbilicaria nylanderiana* (Gulluce et al. 2006) thalli were used for extraction with methanol. Additionally, substances from *Cladonia rangiformis* were extracted by chloroform, methanol and water (Yücel et el. 2007).

Potential of extracted lichen secondary metabolites against *Aspergillus ustus.*

The only lichen tested was the epiphytic species *Hypotrachyna cirrhata*, the substances extracted with water completely inhibiting the germination of *Aspergillus ustus* spores (SGI: 100% for the concentration at 80 µl ml^−1^) (Shahi et al. 2003).

### Analyses of isolated lichen secondary metabolites

#### Potential of extracted lichen secondary metabolites against *Aspergillus flavus*

34 secondary metabolites of lichens grouped in 10 biochemical classes (Table 6) have been tested against *Aspergillus flavus*. The MIC inhibitory potential of the secondary metabolites was 8 × 10^–3^–12.5 mg ml^−1^. The most potent inhibitor tested by Sasidharan et al. (2014) protolichesteric acid (8 × 10^–3^ mg ml^−1^) produced a distinct inhibition zone (21 mm). Diffractaic acid (8.3 × 10^–3^–0.25 mg ml^−1^) proved to be slightly less effective, followed by the inhibitory potentials as follows: orsellinic acid (9.8 × 10^–3^ mg ml^−1^), lecanoric acid (12.6 × 10^–3^–1 mg ml^−1^), norstictic acid (13.8 × 10^–3^–0.5 mg ml^−1^), protocetraric acid (14.1 × 10^–3^–1 mg ml^−1^), 2-hydroxy-4-methoxy-3,6-dimethylbenzoic acid (16 × 10^–3^ mg ml^−1^ MIC, 23 mm IZ), usnic acid (20.1 × 10^–3^–0.5 mg ml^−1^ MIC, 17 mm IZ), atranorin (32 × 10^–3^–1 mg ml^−1^ MIC, 18 mm IZ) and barbatolic acid (0.1 mg ml^−1^). The inhibition potential determined in the remaining data is above 0.25 mg ml^−1^ MIC (Table 6).

Measurement of mycelium growth over time (PFT) shows a high inhibitory effect induced by metabolites belonging to monocyclic aromatic derivatives, methyl orsellinate (IR: 80%), and its derivative from the β-pathway (IR: 90%). Lobaric acid (orcinol depsidone) limits mycelium growth at the same level, in contrast to lecanoric acid (orcinol depside), which inhibitory potential was as twice as weaker. No effects were caused by erythrin, sekikaic and thamnolic acids (Table 6).

#### Potential of extracted lichen secondary metabolites against *Aspergillus fumigatus*

17 lichen secondary metabolites grouped in nine biochemical classes (Table 7) have been tested against *Aspergillus fumigatus* in the MIC range of 0.125–12.5 mg ml^−1^. The secondary metabolite with the highest MIC potential was lecanoric acid, followed by weaker concentrations starting at 0.25 mg ml^−1^ by isodivaricatic acid; a derivative of this compound, divaricatic acid, proved to be the weakest. The inhibitory potential of the other secondary metabolites starts with a concentration at 0.4 mg ml^−1^. A concentration of 250 µg 41.7 µl^−1^ MIC is reported for salazinic acid (Table 7).

The IZ value for methyl haematommate did not show any inhibitory effects, while barbatolic acid was effective as an inhabitant of fungal sporulation (IS) (Table 7).

#### Potential of extracted lichen secondary metabolites against *Aspergillus nidulans*

Six secondary metabolites of lichens grouped into five biochemical classes were investigated against *Aspergillus nidulans* according to the MIC method. The strongest inhibition was exerted by norstictic acid (6.4 × 10^–3^ mg ml^−1^) compared to the weakest effect of usnic acid (14.3 × 10^–3^ mg ml^−1^) (Table S8).

#### Potential of extracted lichen secondary metabolites against *Aspergillus niger*

Against *Aspergillus niger*, 37 secondary metabolites of lichens grouped in nine biochemical classes (Table 8) were investigated. The inhibitory potential of all tested combinations reached a maximum at a concentration of 1 mg ml^−1^. The strongest (3.9 × 10^–3^ mg ml^−1^) inhibition among the tested combinations according to MIC was exerted by the lecanoric acid; however, usnic, protocetraric, diffractaic, orsellinic and norstictic acids were slightly weaker. Belonging to anthraquinone class, parietin, parietinic acid, fallacinol, fallacinal and emodin showed an inhibitory effect at a higher concentration, and the effectiveness of salazinic acid was determined at 250 µg 41.7 µl^−1^. The MIC efficacy for the other secondary metabolites was determined at higher concentrations. Olivetoric acid, chloroatranorin, barbatolic acid and thamnolic acid were ineffective, as well as methyl haematommate according to IZ (Table 8).

The percentage inhibition of the mycelium (according to the DDM method) by several compounds from the anthraquinone class was relatively weak (IR: 13–38%) (Table 8).

#### Potential of extracted lichen secondary metabolites against *Aspergillus ochraceus*

Six secondary metabolites of lichens grouped into five biochemical classes were investigated against *Aspergillus ochraceus* according to the MIC method, of which diffractaic acid inhibited the strongest (5.2 × 10^–3^ mg ml^−1^) compared to the weakest activity of orsellinic acid (14.5 × 10^–3^ mg ml^−1^) (Table S9).

#### Potential of extracted lichen secondary metabolites against *Aspergillus parasiticus*

The inhibitory potential of eight metabolites of secondary lichens grouped into six biochemical classes were tested against *Aspergillus parasiticus* according to the MIC method and ranged from 5.9 × 10^–3^ mg ml^−1^ for diffractaic acid to 400 × 10^–3^ mg ml^−1^ for barbatolic acid. No effect was found for thamnolic acid (Table S10).

## Discussion

### General issues for lichen extracts

The systematic analysis of the literature data indicates that the results were mainly based on the determination of the MIC and IZ. A comparison of the inhibitory potential against the tested *Aspergillus* species is possible, especially in the case of the known inhibitory effects for both complementary methods of measuring results. If the researchers chose to specify the biocidal concentration (MFC), it determines the limit to which the growth of the fungus can be preserved despite unfavourable conditions. The measured inhibition values for other methods are of marginal importance due to their low frequency and the inability to directly compare them with the MIC or IZ (for example, the percentage of inhibition with the inhibitory concentration).

Different organic solvents (mainly acetone and methanol) with varying degrees of polarity were used for the extraction of lichen compounds. Water was a secondary solvent, but in the context of the use of lichen extracts as fungicidal agents in, for example, buildings or agriculture, more research is required on the effectiveness of water extraction methods and the effect of water-extracted lichen substances on *Aspergillus* species. From an ecological point of view, the collected data have uncertain findings due to the adoption of methodological assumptions, most of which do not correspond to the possible environmental conditions.

### Inhibitory potential of crude extracts from lichens against *Aspergillus flavus*

As regards the inhibitory effect of extracted compounds from a lichen thallus, the greatest attention was paid to preventing the growth of *Aspergillus flavus*, *A. fumigatus* and *A. niger*. Compounds extracted from 72 species of lichens of all ecological groups were investigated against *A. flavus*. Those extracted with methanol in the Soxhlet apparatus for 72 h from the epigeic species *Cladonia foliacea* in the proportions of 10 g of thallus/250 ml of solvent were highly effective (15.62 × 10^–3^ mg ml^−1^) (Aslan et al. 2006). A similar combination was used by Mitrović et al. (2011), who extracted the same proportions of *C. foliacea* and methanol at room temperature for 24 h but dissolved the obtained substances in dimethyl sulfoxide (DMSO), which seems to be the reason for the lower activity of the tested combination. A weaker inhibition was shown by compounds extracted from *Pseudevernia furfuracea* with methanol (0.04 mg ml^−1^ MIC) obtained in the same way as Mitrović et al. (2011), but without dissolution in DMSO; the effective MFC concentration of these substances, including the acetone-extracted ones from *Platismatia glauca* (Mitrović et al. 2014), was higher than those of most combinations tested against *A. flavus* in the literature data (cf. Table 1, Table S3, S4). At the strongest MIC concentration levels, the acetone extract from *P. furfuracea*, prepared analogously to the methanol one in the study by Mitrović et al. (2014), plays an important role. The substances extracted from epilithic lichens turned out to be much weaker than representatives of epiphytic and epigeic species in this respect.

The temperature differences in the process of extracting compounds from *Cladonia foliacea* were probably one of the reasons for the observed difference in value of fungal inhibition (increased temperature in the Soxhlet apparatus *vs* room temperature). Furthermore, the concentration could be dependent on the time difference in the extraction process and the dissolution of the extracted substances in organic solvents and DMSO through possible differences in polarity between the solvents. This may result in a difference not only in obtaining the absolute concentration of compounds in the extract, but also different proportions between the biochemical groups, regardless of the environmental (Armaleo et al. 2008; Neupane et al. 2017) and nutritional (Fazio et al. 2014; Santiago et al. 2020) influences or ecotype (Norouzi et al. 2020) on the concentration of substances in the lichen thalli.

The values of the IZ obtained by the tested combinations, despite achieving a diameter of > 20 mm, in comparison to the obtained MIC values, do not play a significant role as an indicator of the antibiotic potential of the lichen mixture compounds against *Aspergillus flavus*. Unlike the water-extracted compounds from *Hypotrachyna cirrhata* and *Leucodermia leucomelos* prepared by maceration of 10 g of thallus in 20 ml of water, which completely inhibited mycelial colony growth (SGI) (Shahi et al. 2001, 2003).

### Inhibitory potential of crude extracts of lichens against *Aspergillus fumigatus*

Lichen substances from 46 lichen species have been tested against *Aspergillus fumigatus*. The analysis shows that the methanol combination used by Mitrović et al. (2011) proved that the effectiveness of the compounds from *Parmelia sulcata* (9.8 × 10^–3^ mg ml^−1^) was weaker in the study by Ranković and Kosanić (2012), despite a similar methodology (50 g of thallus was extracted in the Soxhlet apparatus and the concentration of extracted substances in DMSO) and Ranković et al. (2007a) (50 g of thallus/250 ml of methanol in a Soxhlet apparatus for 7 h; the tested concentration of the extracted substances was determined in DMSO). It seems, from the methodological point of view, that this may result from the variability of the content of lichen substances in the lichen thalli (polysaccharides: Akbulut and Yildiz 2010; Boustie et al. 2011; secondary metabolites: Prokopyev et al. 2018) subjected to extraction, which determines the differences in specific compound concentrations, even if dissolved at a determined concentration, and thus different interactions between them (Furmanek Ł., Czarnota P. and Seaward M. R. D., unpublished data).

Even more effective antibiotic activity (3.13 × 10^–1^ mg ml^−1^) was demonstrated for the substances extracted with methanol from *Cladonia foliacea* (Mitrović et al. 2011). On the other hand, extracts with the fungicidal effectiveness obtained from *Nephroma arcticum* were prepared in a Soxhlet apparatus (5 g of thallus/100 ml of water extracted for 3 h) (Land and Lundström 1998).

Extracts from epilithic lichens showed a relatively weak antifungal potential, apart from the 30 mm diameter of IZ obtained for the ethanol-extracted compounds from *Umbilicaria polyphylla* (Ranković et al. 2009).

### Inhibitory potential of crude extracts of lichens against *Aspergillus niger*

The *Aspergillus niger* mycelium has been subjected to substances extracted from 48 lichen species. Those from epiphytic *Hypogymnia physodes* inhibited the mycelium at the equivalent concentration as the compounds extracted from *Parmelia sulcata* (Mitrović et al. 2011) in the case of *A. fumigatus*. Against *A. niger*, the combinations were tested by Manojlović et al. (2012a) using *Umbilicaria cylindrica* thalli extracted at room temperature (500 g thallus/2000 ml methanol and chloroform for three days), as well as by the Aslan et al. (2006) with substances extracted from *Evernia prunastri* using MIC and IZ (6 mm in diameter; concentration: 30 mg ml^−1^; dose: 300 µg/disk). Unknown proportions of *Variospora dolimiticola* thallus and ethanol in the Soxhlet apparatus for 24 h allowed the extraction of a relatively effective mixture of lichen compounds (Manojlović et al. 2002).

### Inhibitory potential of crude extracts from lichens against other *Aspergillus* species

The methodology of extract preparation used by Mitrović et al. (2011) indicates the potential use of crude extracts from *Evernia prunastri*, *Hypogymnia physodes* and *Parmelia sulcata* against *Aspergillus restrictus*, as well as water-based ones from *Hypotrachyna cirrhata* against *A. candidus* and *A. ustus*, and *H. cirrhata* (Shahi et al. 2003) and *Leucodermia leucomelos* against *A. parasiticus* (Shahi et al. 2001). Four species, *A. candidus*, *A. restrictus*, *A. stellatus* and *A. ustus* that were resistant to all investigated combinations require further investigation.

### Inhibitory potential of lichen extracts—important factors and interpretation problems.

Lichen secondary metabolites that have a significant inhibitory effect on the tested species are presented in Table S11. The above-mentioned most promising combinations were prepared at an increased temperature (Soxhlet apparatus) or at room temperature. The high efficiency of both these different extraction methods emphasise the potential of the crude extracts of lichens regardless of temperature, but in relation to the chemical composition of the extracted substances and their concentration.

Methanol proved to be the solvent capable of obtaining the most extracts of antibiotic nature; less effective were acetone, ethanol, chloroform and water. The water-solvent levels for most of the tested combinations were too weak to obtain a threshold concentration to induce mycelial inhibition. However, the water-soluble lichen compounds extracted from *Hypotrachyna cirrhata* and *Leucodermia leucomelos* require more attention in future research.

Among all the tested combinations based on crude extracts, only a relatively small number (11 species; Tables 1, 2, 3, 4, 5, Tables S3, S4, S5, S6, S7) have a stronger inhibitory potential, determined mainly on the basis of the MIC, which results from a more significant importance of the MIC value than the IZ (Furmanek et al. 2019). From the point of view of those species belonging to particular ecological groups, the most important are epiphytic macrolichens (*Evernia prunastri*, *Hypogymnia physodes*, *Hypotrachyna cirrhata*, *Leucodermia leucomelos*, *Parmelia sulcata*, *Platismatia glauca* and *Pseudevernia furfuracea*). As regards obtaining antibiotic compounds, the commonly occurring *Hypogymnia physodes*, *Parmelia sulcata* and *Pseudevernia furfuracea* are of the greatest importance. In contrast to epiphytes, the group of epigeic (*Cladonia foliacea*, *Nephroma arcticum*) and epilithic species (*Umbilicaria cylindrica*, *Variospora dolomiticola*) constitute a small part of the investigated lichen species against *Aspergillus* fungi.

It would appear that the difference in the antibiotic potential of the lichen compounds of species of different ecological groups results not only from the greater number of representatives of epiphytic species tested, but also from the difference in the concentration of the lichen substances biosynthesized by them, with a predominance for epiphytic lichens and their easier extraction from the thallus in relation to epigeic and epilithic species. The general antifungal potential of the extracted lichen compounds will be the result of the sum of interactions between them (antagonism of lichen substances: Kowalski et al. 2011); therefore, the strong inhibitory potential of individual compounds present in the extract (mainly secondary metabolites) is of less importance for the analysis of the crude extracts.

Against *Aspergillus flavus*, the compounds present in the extracts from five lichen species (*Cladonia foliacea*, *Hypotrachyna cirrhata*, *Leucodermia leucomelos*, *Platismatia glauca* and *Pseudevernia furfuracea*) were of the greatest importance, in which, depending on the species, several secondary metabolites were recognized (Table S11).

Substance mixtures extracted from *Evernia prunastri*, *Hypogymnia physodes*, *Umbilicaria cylindrica* and *Variospora dolomiticola* are important in inhibiting the growth of *Aspergillus niger* mycelium. *A. fumigatus* was most susceptible to a mixture of substances from the lichens *Cladonia foliacea*, *Nephroma arcticum* and *Parmelia sulcata*, as was *Aspergillus restrictus* for compounds from *Evernia prunastri*, *Hypogymnia physodes* and *Parmelia sulcata*. Differences between mold and lichen species indicate (for at least the three most-investigated species *A. flavus*, *A. fumigatus* and *A. niger*) specific interactions between the tested lichen extracts against *Aspergillus* species.

### Inhibitory potential—crude extracts *vs* nonlichen reference substances

The lack of a standard assessment of the inhibitory effects of lichen compounds mixtures against fungi (Furmanek et al. 2019) could be compared to the potential of antifungal reference substances (Table S12A-B).

Comparison of the antifungal potential of lichen substances against *Aspergillus flavus* at the MIC level in the range of 15.62 × 10^–3^ to 0.63 mg ml^−1^ (MIC below 1 mg ml^−1^) for, respectively, methanol-extracted substance mixtures from *Cladonia foliacea* (Aslan et al. 2006) and *Parmelia sulcata* (Mitrović et al. 2014), and other combinations with MICs within this range (see: Table 1, Tables S3, S4) to the reference substances MIC values allow us to conclude that the crude extracts can compete with amphotericin B, ketoconazole, clotrimazole, fluconazole, amoxycilin, cefazolin, chloramphenicol, moxiphloxacin, tobramycin and benzalkonium chloride. However, the antifungal potential of itraconazole, voriconazole, posaconazole, ravuconazole, anidulafungin, micafungin and caspofungin is still more effective than the antibiotic effect of lichen extracts. The mixture of compounds extracted with methanol from *Pseudevernia furfuracea* and *Platismatia glauca* (Mitrović et al. 2014) showed even greater biocidal efficiency (MFC) compared to fluconazole and chloramphenicol, although weaker than ketoconazole and benzalkonium chloride. Interestingly, the analysis of lichen crude extracts against *Aspergillus flavus* showed a similar level of antifungal potential for most tested extracts based on IZ compared to amphotericin B and fluconazole, but voriconazole is relatively more effective (cf. Table 1, Tables S3, S4, S12A).

The comparison [for the range [1] < 9.8 × 10^–3^ to [2] 3.13 × 10^–1^ mg ml^−1^, for methanol-extracted compounds from [2] *Cladonia foliacea* (MIC) and [1] *Parmelia sulcata* (MIC, MFC) (Mitrović et al. 2011)] with the MIC of the reference substances against *Aspergillus fumigatus* indicates a similar level of inhibition compared to ketoconazole, itraconazole and fluconazole. Amphotericin B and voriconazole were more effective, while posaconazole, ravuconazole, anidalafungin, micafungin and caspofungin have the clearest effectiveness. The only reported MFC value for fluconazole is weaker compared to the combination with *Parmelia sulcata* (Mitrović et al. 2011). The reports of IZ values in the literature data, as in the case of *A. flavus*, in most of the tested combinations (regardless of methodological differences), are similar to the results of the reference substances (ketoconazole, nystatin and fluconazole) (cf. Tables 2, 3, Tables S5, S12A).

The potential range for methanol-extracted compounds from *Hypogymnia physodes* (MIC, MFC) and extracted by ethanol from *Variospora dolomiticola* (Manojlović et al. 2002), as well as by methanol and water (5 × 10^–3^ mg ml^−1^ MIC) from the unrecognized species of *Peltigera* sp. (Bisht et al. 2014) is in the range of < 9.8 × 10^–3^—160 × 10^–3^ mg ml^−1^ against *Aspergillus niger*. The values are comparable to the effectiveness of amphotericin B, ketoconazole, clotrimazole and fluconazole, slightly weaker compared to itraconazole and voriconazole, and clearly weaker compared to posaconazole, anidalafungin, micafungin and caspofungin. The only available MFC literature data value for fluconazole is clearly weaker than the extracted mixture of compounds from *Hypogymnia physodes* (Manojlović et al. 2002). The IZ values of the extracts compared to those obtained by reference substances, similarly to *Aspergillus flavus* and *A. fumigatus*, in most cases are partially competitive for ketoconazole, clotrimazole, nystatin and fluconazole (cf. Table 4, Tables S6, S7, S12A).

The methanol-extracted compounds from *Parmelia sulcata* tested against *Aspergillus restrictus* (Mitrović et al. 2011) proved to be slightly weaker than the MIC value of fluconazole, but more effective compared to the MFC value of this reference substance (cf. Table 5, Table S12B).

The analysis of the potential of the extracts does not provide comparable data when compared to the reference substances against *Aspergillus stellatus*. Due to the adoption of a different research methodology for the poorly investigated lichen crude extracts and reference substances against *Aspergillus ustus*, *A. candidus* and *A. parasiticus*, no comparative analysis of the effectiveness of the tested combinations was undertaken.

### Inhibitory potential—lichen secondary compounds *vs* nonlichen reference substances

The relatively small number of the tested crude extracts with the antifungal potential comparable to the potential of the antibiotics presented in Table S12A-B is explained by the antagonistic interactions between the extracted compounds present in the tested extracts. From this point of view, the tested individual lichen secondary metabolites should have a higher potency against fungi in general, and not only those from the genus *Aspergillus*. On the other hand, the high fungistatic efficiency of one of the metabolites does not need to transform directly into the high antifungal potential of the tested substance mixture, due to the relationship with other compounds in extract. The above hypothesis is probably confirmed by the analysis of the effectiveness of lichen secondary metabolites, which generally inhibited the tested mycelium at a lower concentration than the mixtures of extracted substances (cf. Tables 1, 2, 3, 4, 5, 6, 7, 8, Tables S3–S10).

The MIC range of secondary metabolites at [1] 8 × 10^–3^ to [2] 0.1 mg ml^−1^, for [1] protolichesterinic acid (Sasidharan et al. 2014) and [2] barbatolic acid (Sarıözlü et al. 2016), as well as secondary metabolites within this potential range (namely atranorin, diffractaic acid, lecanoric acid, norstictic acid, orsellinic acid, protocetraric acid, 2-hydroxy-4-methoxy-3,6-dimethylbenzoic acid and usnic acid) against *Aspergillus flavus* compared to the potential of reference substances is partially convergent (amphotericin B, ketoconazole, clotrimazole, itraconazole, voriconazole, fluconazole, amoxycillin, cefazolin, chloramphenicol, moxifloxacin, tobramycin and benzalkonium chloride). By extending the activity range of secondary metabolites to 1 mg ml^−1^, most of the secondary metabolites listed in Table 6 would be achieved the potential of amoxicillin, cefazoline, chloramphenicol, moxifloxacin and tobramycin. However, posaconazole, ravuconazole, anidulafungin, micafungin and caspofungin were more effective. Values of IZ of secondary metabolites in the range of 17–23 mm for ( +)-usnic acid, atranorin, protolichesterinic acid and 2-hydroxy-4-methoxy-3,6-dimethylbenzoic acid are similar to amphotericin B and ketoconazole, but weaker than voriconazole (cf. Table 6, Table S12A).

The MIC values of metabolites tested against *Aspergillus fumigatus* shows that lecanoric acid tested in the Ranković and Mišić (2008) experiment is comparable to fluconazole (cf. Table 7, Table S12A), excluding the potential of salazinic acid (250 µg 41.7 µl^−1^) (Candan et al. 2007).

*Aspergillus niger* was more susceptible to the inhibitory potential of secondary metabolites compared to *A. flavus* and *A. fumigatus*, which in the MIC range of 3.9 × 10^–3^–40 × 10^–3^ mg ml^−1^ (orsellinic acid, lecanoric acid, diffractaic acid, protocetraric acid, norstictic acid, parietin, parietinic acid, fallacinol, fallacinal, emodin and usnic acid) overlaps in part with amphotericin B, ketoconazole, clotrimazole, itraconazole and fluconazole, but are weaker than posaconazole, anidulafungin, micafungin and caspofungin. Salazinic acid (250 µg 41.7 µl^−1^) also presents an important inhibitory potential in the study of Candan et al. (2007) (cf. Table 8, Table S12A).

Poorly studied secondary lichen metabolites against *Aspergillus parasiticus* provide potentially stronger antibiotic compounds in the form of orsellinic, lecanoric, diffractaic, protocetraric, norstictic and usnic acids, which are more potent compared to the MIC of ketoconazole and clotrimazole (cf. Tables S10, S12B); on the other hand, these acids appear to be stronger than the MIC potential of clotrimazole used against *Aspergillus nidulans*, but less effective than amphotericin B, itraconazole, voriconazole, posaconazole, caspofungin, micafungin and anidulafungin (cf. Tables S8, S12B). Similar conclusions are drawn for *A. ochraceus*, against which the tested secondary metabolites (the same as against *A. nidulans*) are stronger than the MIC potential of clotrimazole, but weaker than itraconazole, voriconazole and posaconazole (cf. Tables S9, S12B).

### What are the most inhibitory secondary metabolites in crude extracts?

The performed analysis of crude extracts and secondary metabolites of lichens confirms the importance of the investigated compounds as effective antifungal agents. The crude extracts are of less importance according to inhibitory effects. Individual secondary metabolites, independent of interactions with other compounds of primary and secondary metabolism, have a greater antibiotic potential.

It can be assumed that the high antifungal potential of the mixtures that showed the highest effectiveness against *Aspergillus* species could be of significance, at least, for atranorin and usnic acid biosynthesized by *Cladonia foliacea*, and methyl β-orsellinate and methyl orsellinate (which achieved high percentages of IR value) by *Platismatia glauca* and *Pseudevernia furfuracea*, atranorin and protocetraric acid by *Hypotrachyna cirrhata*, and at least salazinic acid by *Leucodermia leucomelos*, which were tested against *A. flavus* (Table S11).

For the mixtures tested against *A. fumigatus*, divaricatic acid in an extract from *Parmelia sulcata* could be of importance for its high inhibitory potential; for *C. foliacea*, this potential could result from uninvestigated single lichen compounds or from interactions synergistically increasing the inhibition of mycelial growth, as in the case of usnic acid in the presence of atranorin and fumarprotocetric acid, rather than from the effectiveness of the three metabolites alone; a similar situation may occur for the potential of *Nephroma arcticum*.

In the case of the extract from *Hypogymnia physodes*, it is difficult to predict which compounds (given in Table S11) could have the greatest importance for the inhibitory potential against *Aspergillus niger* due to the lack of literature data for the tested individual lichen secondary metabolites. In the case of the *Evernia prunastri* crude extract, this role may have been caused by usnic acid, orsellinic acid, and possibly methyl orsellinate. The inhibitory potential of extracted compounds from *Umbilicaria cylindrica* could be determined by the presence of norstictic and usnic acids, but the influence of salazinic acid and methyl β-orcinocarboxylate cannot be excluded. On the other hand, the presence of parietin, parietinic acid, fallacinol, fallacinal and emodin could be the cause of the extract potential obtained from *Variospora dolomiticola*, simultaneously showing antagonistic interactions between extracted compounds in an obtained extract.

Protocetraric acid is the only comparable secondary metabolite from the *Hypotrachyna cirrhata* extract against *Aspergillus parasiticus* that could influence the inhibitory potential.

There are insufficient data to compare the effectiveness of secondary metabolites to compounds included in extract obtained from *Leucodermia leucomelos* tested against *A. parasiticus*.

This analysis does not provide sufficient data to compare the inhibitory potential of the secondary metabolites present in extracts tested against *Aspergillus ustus*, *A. candidus* and *A. restrictus*.

### Future prospects and problems to solve

Summing up, the inhibitory potential of the crude extracts and lichen secondary metabolites based solely on the IZ would lead to an erroneous conclusion concerning the high efficiency of most of the tested combinations. This error can be avoided by comparing the IZ value with the MIC potential, which reduces the probability of accepting a false sensitivity error (Furmanek et al. 2019). The limited amount of data on the IZ or their small diameter necessitated the interpretation of this analysis mainly on the basis of the MIC potential.

The high potential of protolichesteric acid against *Aspergillus flavus* (Sasidharan et al. 2014) is also confirmed by the antibiotic potential against *Trichophyton rubrum* according to analysis carried out by Furmanek et al. (2019). This effectiveness may be due to the disturbance of the membrane permeability function (Kosanić and Ranković 2019) in contact with the fatty acid (see: Table S2). In addition, the high inhibitory efficiency of secondary metabolites against species of *Aspergillus* is not related to their general chemical structure (belonging to the chemical class), but rather proves the presence of specific functional groups that affect antifungal activity, such as the -OH groups of aromatic substances or -CH_3_ groups attached to annular compounds (Bruchajzer 2017).

The analysis shows that in the case of the well-investigated *Aspergillus flavus* and *A. niger* and the less well-studied *A. parasiticus* and *A. nidulans*, there is a possibility of replacing the pharmacological agents (Table S12A-B), especially ketoconazole and clotrimazole by means of lichen extracts and their secondary metabolites. Against *A. fumigatus*, fluconazole antibiotic may be replaced. However, the potential of posaconazole, ravuconazole, anidulafungin, micofungin and caspofungin is still higher than antifungal potential of extracts and secondary metabolites of lichens.

As regards future experiments against *Aspergillus*, research on weaker investigated species (*A. candidus*, *A. nidulans*, *A. ochraceus*, *A. parasiticus*, *A. rastrictus*, *A. stellatus* and *A. ustus*) should be extended with the use of compound mixtures and especially individual secondary metabolites of lichens, which show a higher inhibitory potential. It would be interesting to use the combined potential of at least two interacting secondary compounds in the context of possible synergistic activity (from the point of view of inhibitory effects), which is particularly important for mixtures with a higher inhibitory potential than the metabolites alone (as in case of *Nephroma arcticum*). It would also be advisable to highlight the inhibitory potential of the tested combinations in the form of an inhibition zone (IZ) as complementary to the primary MIC and MFC values. For the determination of ecological relationships, the PFT method and greater emphasis on water extraction methods are recommended.

The conclusions resulting from this analysis indicate that it is possible to use several tested mixtures of extracted compounds and at least a dozen secondary metabolites as potential plant protection agents, food preservatives, and drugs against mycoses and mycotoxicoses, as well as indoor antifungal agents. The safety and efficacy of the most effective combinations should be subjected to further field and clinical trials.

## Conclusions

1. Crude extracts and individual secondary metabolites may constitute novel antibiotic agents against *Aspergillus* species. The most investigated *Aspergillus* species are *A. flavus*, *A. fumigatus* and *A. niger.* Further research is necessary which focuses on poorly investigated *Aspergillus* species using MIC and IZ values.
2. The most promising crude extracts were obtained from macrolichen thalli such as *Cladonia foliacea*, *Evernia prunastri*, *Hypogymnia physodes*, *Hypotrachyna cirrhata*, *Leucodermia leucomelos*, *Nephroma arcticum*, *Parmelia sulcata*, *Platismatia glauca*, *Pseudevernia furfuracea*, *Umbilicaria cylindrica* and *Variospora dolomiticola.*
3. Generally, among secondary metabolites, the most inhibitory effects were exerted by lecanoric, orsellinic and protolichesterinic acids. It is possible to exploit the antifungal potential of a broader set of lichen secondary metabolites in relation to individual mold species. In the case of poorly studied *Aspergillus* species, the literature on the subject does not provide a significant amount of data on the potential of reference substances, which makes the relative assessment of the antibiotic potential of lichen compounds difficult.
4. In future, ketoconazole and clotrimazole might be replaced by lichen compounds against *A. flavus*, *A. nidulans A. niger* and *A. parasiticus*, and fluconazole in the case of *A. fumigatus*. The potential of lichen substances, especially of individual secondary metabolites, against a particular species of *Aspergillus* overlaps with the level of antifungal potential of the reference substances for a wider set of compounds. However, the antifungal potential of posaconazole, ravuconazole, anidulafungin, micafungin and caspofungin is greater.
5. The necessity to use some new methodological combinations such as crude extracts extracted from at least two species of lichens in one extract or a mixture of individual lichen metabolites to understand the interactions between the various compounds.
6. The necessity to use a PFT as a measurement method for mycelium growth and to use water more frequently as an extraction solvent in an ecological context. The authors recommend the use of methods of water extraction in the future; as well as approaches to increase the effectiveness of this process and obtain higher concentrations of lichen compounds in extracts.
7. Field and clinical trials for lichen compounds against *Aspergillus* species should be undertaken.

## Supplementary Information

Below is the link to the electronic supplementary material.

Supplementary file1 (DOCX 77 KB)
