# Supplementary material for: The effect of lichen secondary metabolites on Aspergillus fungi
Source: Arch Microbiol. 2021 Dec 29;204(1):100. doi: 10.1007/s00203-021-02649-0 (PMC8716355; doi:10.1007/s00203-021-02649-0)
Supplement: Supplementary file 1 — Supplementary file1 (DOCX 77 KB) [file 203_2021_2649_MOESM1_ESM.docx]

Tab. S1. Lichen species assigned to the main ecological groups tested on their antifungal potential against *Aspergillus* spp. in the form of lichen extracts.

| **Ecological group** | **Lichen species** |
| --- | --- |
| **Epiphytic lichens** | *Alectoria sarmentosa* (Ach.) Ach. |
|  | *Anaptychia ciliaris* (L.) Körb. ex A. Massal. |
|  | *Bulbothrix setschwanensis* (Zahlbr.) Hale |
|  | *Dirinaria consimilis* (Stirt.) D.D. Awasthi |
|  | *Evernia divaricata* (L.) Ach. |
|  | *Evernia prunastri* (L.) Ach. |
|  | *Flavoparmelia caperata* (L.) Hale  [= *Parmelia caperata* (L). Ach.] |
|  | *Heterodermia diademata* (Taylor) D.D. Awasthi |
|  | *Heterodermia obscurata* (Nyl.) Trevis. |
|  | *Hypogymnia physodes* (L.) Nyl. |
|  | *Hypotrachyna cirrhata* (Fr.) Divakar, A. Crespo, Sipman, Elix & Lumbsch  [= *Parmelia cirrhata* Fr.] |
|  | *Hypotrachyna nepalensis* (Taylor) Divakar, A. Crespo, Sipman, Elix & Lumbsch  [= *Everniastrum nepalense* (Taylor) Hale ex Sipman] |
|  | *Letharia vulpina* (L.) Hue |
|  | *Leucodermia leucomelos* (L.) Kalb.  [= *Heterodermia leucomelos* (L.) Poelt] |
|  | *Menegazzia terebrata* (Hoffm.) A. Massal.  [= *Parmelia pertusa* Schaer.] |
|  | *Ochrolechia androgyna* (Hoffm.) Arnold |
|  | *Parmelia omphalodes* (L.) Ach. |
|  | *Parmelia saxatilis* (L.) Ach. |
|  | *Parmelia sulcata* Taylor |
|  | *Parmeliopsis ambigua* (Hoffm.) Nyl. |
|  | *Parmeliopsis hyperopta* (Ach.) Vain. |
|  | *Parmotrema andinum* (Müll. Arg.) Hale |
|  | *Parmotrema crinitum* (Ach.) M. Choisy  [= *Parmelia crinita* Ach.] |
|  | *Parmotrema grayanum* (Hue) Hale |
|  | *Parmotrema perlatum* (Huds.) M. Choisy |
|  | *Parmotrema praesorediosum* (Nyl.) Hale |
|  | *Parmotrema reticulatum* (Taylor) M. Choisy  [= *Parmelia reticulata* Taylor] |
|  | *Parmotrema thomsonii* (Stirt.) A. Crespo, Divakar & Elix  [= *Parmelaria thomsonii* (Stirt.) D.D. Awasthi] |
|  | *Parmotrema tinctorum* (Despr. ex Nyl.) Hale |
|  | *Physcia aipolia* (Ehrh. ex Humb.) Fürnr. |
|  | *Platismatia glauca* (L.) W.L. Culb. & C.F. Culb. |
|  | *Protousnea poeppigii* (Nees & Flot.) Krog |
|  | *Pseudevernia furfuracea* (L.) Zopf |
|  | *Pseudevernia furfuracea* var. *ceratea* (Ach.) D. Hawksw. |
|  | *Ramalina conduplicans* Vain. |
|  | *Ramalina farinacea* (L.) Ach. |
|  | *Ramalina hossei* Vain. |
|  | *Ramalina pacifica* Asahina |
|  | *Ramalina pollinaria* (Westr.) Ach. |
|  | *Ramalina polymorpha* (Lilj.) Ach. |
|  | *Teloschistes flavicans* (Sw.) Norman |
|  | *Usnea antiqua* Swinscow & Krog |
|  | *Usnea barbata* (L.) F.H. Wigg. |
|  | *Usnea complanata* (Müll. Arg.) Motyka |
|  | *Usnea florida* (L.) F.H. Wigg. |
|  | *Usnea maculata* Stirt. |
|  | *Usnea sanguinea* Swinscow & Krog |
|  | *Usnea submollis* J. Steiner |
|  | *Vulpicida pinastri* (Scop.) J.-E. Mattsson & M.J. Lai  [= *Cetraria pinastri* (Scop.) Gray] |
| **Epigeic lichens** | *Cetraria islandica* (L.) Ach. |
|  | *Cladonia digitata* (L.) Hoffm. |
|  | *Cladonia fimbriata* (L.) Fr. |
|  | *Cladonia foliacea* (Huds.) Willd. |
|  | *Cladonia furcata* (Huds.) Schrad. |
|  | *Cladonia pyxidata* (L.) Hoffm. |
|  | *Cladonia rangiferina* (L.) Weber ex. F.H. Wigg. |
|  | *Cladonia rangiformis* Hoffm. |
|  | *Cladonia* sp. |
|  | *Cladonia subulata* (L.) Weber ex F.H. Wigg. |
|  | *Gyalolechia fulgens* (Sw.) Søchting, Frödén &Arup  [=*Fulgensia fulgens* (Sw.) Elenkin] |
|  | *Nephroma arcticum* (L.) Torss. |
|  | *Peltigera aphthosa* (L.) Willd. |
|  | *Peltigera* sp. |
|  | *Stereocaulon paschale* (L.) Hoffm. |
| **Epilithic lichens** | *Acarospora fuscata* (Ach.) Arnold. |
|  | *Arctoparmelia centrifuga* (L.) Hale  [= *Parmelia centrifuga* (L.) Ach.] |
|  | *Aspicilia cinerea* (L.) Körb. |
|  | *Caloplaca cerina* (Hedw.) Th. Fr. |
|  | *Dermatocarpon miniatum* (L.) W. Mann |
|  | *Lasallia pustulata* (L.) Mérat |
|  | *Lathagrium cristatum* (L.) Otálora, P.M. Jørg. & Wedin  [= *Collema cristatum* (L.) Weber ex F.H. Wigg.] |
|  | *Lecanora frustulosa* (Dicks.) Ach. |
|  | *Nephroma parile* (Ach.) Ach. |
|  | *Ochrolechia parella* (L.) A. Massal. |
|  | *Ochrolechia tartarea* (L.) A. Massal. |
|  | *Physcia caesia* (Hoffm.) Fürnr. |
|  | *Protoparmeliopsis muralis* (Schreb.) M. Choisy  [= *Lecanora muralis* (Schreb). Rabenh.] |
|  | *Tephromela atra* (Huds.) Hafellner  [=*Lecanora atra* (Huds.) Ach.] |
|  | *Thalloidima candidum* (Weber) A. Massal.  [= *Toninia candida* (Weber) Th. Fr.] |
|  | *Umbilicaria crustulosa* (Ach.) Lamy |
|  | *Umbilicaria cylindrica* (L.) Delise |
|  | *Umbilicaria nylanderiana* (Zahlbr.) H. Magn. |
|  | *Umbilicaria polyphylla* (L.) Baumg. |
|  | *Variospora dolomiticola* (Hue) Arup, Søchting & Frödén  [= *Caloplaca schaereri* (Flörke) Zahlbr.] |
|  | *Xanthoparmelia pulla* (Ach.) O. Blanco, A. Crespo, Elix, D. Hawksw. & Lumbsch  [= *Neofuscelia pulla* (Ach.) Essl.] |
|  | *Xanthoparmelia xanthomelaena* (Müll. Arg.) Hale.  [= *Parmelia arseneana* Gyeln.] |

Tab. S2. Biochemical classes of lichen secondary metabolites used against species of *Aspergillus*. Unless otherwise stated, the names of the biochemical classes of secondary metabolites are given according to Elix (2014).

| **Biochemical classes of lichen secondary metabolites** | **Secondary metabolites** |
| --- | --- |
| aliphatic acids | protolichesterinic acid |
| anthraquinones | emodin  erythroglaucin  fallacinal  fallacinol (teloschistin)  parietinic acid  physcion (parietin)  xanthorin |
| benzyl esters | barbatolic acid |
| monocyclic aromatic derivatives | ethyl orsellinate  methyl haematommate  methyl orsellinate  methyl β-orsellinate (methyl β-orcinolcarboxylate)  orsellinic acid |
| orcinol depsides | 2'-O-methylanziaic acid  divaricatic acid  erythrin  evernic acid  isodivaricatic acid^†^  lecanoric acid  olivetoric acid  sekikaic acid  sphaerophorin |
| β-orcinol depsides | 2-hydroxy-4-methoxy-3,6-dimethylbenzoic acid^††^  atranorin  chloroatranorin  diffractaic acid  thamnolic acid |
| orcinol depsidones | lobaric acid  physodic acid  variolaric acid  α-collatolic acid |
| β-orcinol depsidones | 1’-chloropannarin^†††^  fumarprotocetraric acid  norstictic acid  pannarin  protocetraric acid  psoromic acid  salazinic acid  stictic acid  vicanicin |
| orcinol tridepsides | gyrophoric acid |
| terpenoids | zeorin (hopane-6α,22-diol) |
| usnic acid derivatives | (+)-usnic acid/usnic acid |

**^†^** (depside: Schmeda-Hirschmann et al. 2008)

**^††^** (depside: Aravind et al. 2014)

**^†††^** (depsidone: Huneck and Lamb 1975)

Tab. S3. Effect of crude extracts from epigeic lichens on *Aspergillus flavus*. Method of measurement abbreviations: BMM = Broth microdilution method (MIC); BTDM = Broth tube dilution method (MIC); DDM= Disk diffusion method (IZ); MMwR = Microdilution method with resazurin (MIC, MFC); AWDM = Agar well diffusion method (IZ). For MIC, MFC and IZ abbreviations: see Tab. 1. Literature abbreviations: [1] Grujičić et al. (2014);
[2] Ranković et al. (2010a); [3] Aslan et al. (2006); [4] Mitrović et al. (2011); [5] Ranković et al. (2009);
[6] Kosanić et al. (2014b); [7] Ranković et al. (2011); [8] Kosanić and Ranković (2011b); [9] Ranković and Mišić (2007); [10] Yücel et al. (2007); [11] Kosanić et al. (2018).

| **Lichen species** | **Extracting solvent** | **Results** | | | **Measurement method** | **Literature** |
| --- | --- | --- | --- | --- | --- | --- |
|  |  | **MIC**  **[**mg·ml^-1^] | **MFC**  **[**mg·ml^-1^] | **IZ** [mm]  (dose) |  |  |
| *Cetraria islandica* | methanol | 5 | ‡ | ‡ | BMM | [1] |
| *Cladonia digitata* | methanol | † | ‡ | † | BTDM; DDM | [2] |
| *Cladonia fimbriata* | methanol | † | ‡ | † | BTDM; DDM | [2] |
| *Cladonia foliacea* | methanol | 15.62 × 10^-3^ | ‡ | 7  (300 µg/disk) | BMM; DDM | [3] |
|  |  | 2.5 | 10 | ‡ | MMwR | [4] |
| *Cladonia furcata* | acetone | 25 | ‡ | 12 (15 µl) | BTDM; DDM | [5] |
|  |  | 25 | ‡ | ‡ | BMM | [6], [7], [8] |
|  | ethanol | 25 | ‡ | 13 (15 µl) | BTDM; DDM | [5] |
|  | methanol | 25 | ‡ | ‡ | BMM | [8] |
|  | water | † | ‡ | † | BTDM; DDM | [5] |
|  |  | † | ‡ | ‡ | BMM | [8] |
| *Cladonia pyxidata* | acetone | 25 | ‡ | ‡ | BMM | [6] |
| *Cladonia rangiferina* | acetone | 12.5 | ‡ | ‡ | BMM | [6] |
|  | ethanol | >200 | † | † | BTDM; AWDM | [9] |
|  | ethyl acetate | >200 | >200 | † |  |  |
|  | water | >200 | † | † |  |  |
| *Cladonia rangiformis* | chloroform | ‡ | ‡ | † | DDM | [10] |
|  | methanol |  |  |  |  |  |
|  | water |  |  |  |  |  |
| *Cladonia subulata* | acetone | 20 | ‡ | ‡ | MMwR | [11] |
| *Gyalolechia fulgens* | methanol | 30 | ‡ | 7 (10 µl) | BTDM; DDM | [2] |

† no effect, ‡ not investigated

Tab. S4. Effect of crude extracts from epilithic lichens on *Aspergillus flavus*. Method of measurement abbreviations: BMM = Broth microdilution method (MIC); BTDM = Broth tube dilution method (MIC); DDM= Disk diffusion method (IZ). For MIC, MFC and IZ abbreviations: see Tab. 1. Literature abbreviations: [1] Kosanić et al. (2014c); [2] Ranković et al. (2010c); [3] Ranković et al. (2007b); [4] Aslan et al. (2006); [5] Ranković et al. (2007a); [6] Kosanić et al. (2016); [7] Ranković et al. (2010b); [8] Kosanić et al. (2010); [9] Ranković et al. (2010a); [10] Ranković et al. (2011); [11] Ranković and Kosanić (2012); [12] Kosanić et al. (2014a); [13] Kosanić and Ranković (2011a); [14] Ranković et al. (2012); [15] Kosanić et al. (2012b); [16] Gulluce et al. (2006);
[17] Ranković et al. (2009); [18] Kosanić and Ranković (2011b).

| **Lichen species** | **Extracting solvent** | **Results** | | | **Measurement method** | **Literature** |
| --- | --- | --- | --- | --- | --- | --- |
|  |  | **MIC [**mg·ml^-1^] | **MFC** | **IZ** [mm] (dose) |  |  |
| *Acarospora fuscata* | acetone | 10 | ‡ | ‡ | BMM | [1] |
| *Arctoparmelia centrifuga* | methanol | † | ‡ | † | BMM, DDM | [2] |
| *Aspicilia cinerea* | acetone | † | ‡ | † | BTDM; DDM | [3] |
|  | methanol |  |  |  |  |  |
|  | water |  |  |  |  |  |
| *Dermatocarpon miniatum* | methanol | ‡ | ‡ | † | BMM; DDM | [4] |
| *Lasallia pustulata* | acetone | 25 | ‡ | 10 (15 µl) | BTDM; DDM | [5] |
|  | methanol | 6.25 | ‡ | 15 (15 µl) |  |  |
|  |  | 10 |  | ‡ | BMM | [6] |
|  | water | † | ‡ | † | BTDM; DDM | [5] |
| *Lathagrium cristatum* | acetone | † | ‡ | † | BTDM; DDM | [3] |
|  | methanol |  |  |  |  |  |
|  | water |  |  |  |  |  |
| *Lecanora frustulosa* | acetone | † | ‡ | † | BTDM; DDM | [7], [8] |
|  | methanol |  |  |  |  |  |
|  | water |  |  |  |  |  |
| *Nephroma parile* | methanol | † | ‡ | † | BMM; DDM | [2] |
| *Ochrolechia parella* | methanol | † | ‡ | † | BTDM; DDM | [9] |
| *Ochrolechia tartarea* | methanol | † | ‡ | † | BMM; DDM | [2] |
| *Physcia caesia* | acetone | † | ‡ | † | BTDM; DDM | [3] |
|  | methanol | 3.12 |  | 20 (15 µl) |  |  |
|  | water | † |  | † |  |  |
| *Protoparmeliopsis muralis* | acetone | † | ‡ | ‡ | BMM | [10] |
|  |  |  |  |  | BTDM; DDM | [11] |
|  | methanol | 25 | ‡ | ‡ | BMM | [12] |
|  |  | † |  | † | BTDM; DDM | [11] |
|  | water | † | ‡ | † | BTDM; DDM | [11] |
| *Tephromela atra* | acetone | 25 | ‡ | ‡ | BMM | [10] |
|  |  | 25 | ‡ | 16 (15 µl) | BTDM; DDM | [11], [13] |
|  |  | 25 | ‡ | 16 (15 µl) |  |  |
|  | methanol | 6.25 | ‡ | 18 (15 µl) |  |  |
|  | water | † | ‡ | † |  |  |
| *Thalloidima candidum* | acetone | 50 | ‡ | ‡ | BMM | [14] |
| *Umbilicaria crustulosa* | acetone | 25 | ‡ | ‡ | BMM | [15] |
|  |  | 25 | ‡ | 10 (15 µl) | BTDM; DDM | [5] |
|  | methanol | 12.5 | ‡ | ‡ | BMM | [12] |
|  |  | 12.5 | ‡ | 16 (15 µl) | BTDM; DDM | [5] |
|  | water | † | ‡ | † |  |  |
| *Umbilicaria cylindrica* | acetone | † | ‡ | † | BTDM; DDM | [5] |
|  |  |  |  | ‡ | BMM | [15] |
|  | methanol | † | ‡ | † | BTDM; DDM | [5] |
|  | water | † | ‡ | † |  |  |
| *Umbilicaria nylanderiana* | methanol | ‡ | ‡ | † | DDM | [16] |
| *Umbilicaria polyphylla* | acetone | 12.5 | ‡ | 14 (15 µl) | BTDM; DDM | [17] |
|  |  | 12.5 | ‡ | ‡ | BMM | [15] |
|  |  | 12.5 | ‡ | ‡ | BMM | [18] |
|  | ethanol | 1.56 | ‡ | 20 (15 µl) | BTDM; DDM | [17] |
|  | methanol | 1.56 | ‡ | ‡ | BMM | [12], [18] |
|  | water | † | ‡ | † | BTDM; DDM | [17] |
|  |  | † | ‡ | ‡ | BMM | [18] |
| *Xanthoparmelia pulla* | methanol | ‡ | ‡ | † | BMM; DDM | [4] |
| *Xanthoparmelia xanthomelaena* | acetone | 5 | ‡ | ‡ | BMM | [1] |

† no effect, ‡ not investigated

Tab. S5. Effect of crude extracts from epilithic lichens on *Aspergillus fumigatus*. Method of measurement abbreviations: BMM = Broth microdilution method (MIC); BTDM = Broth tube dilution method (MIC); DDM = Disk diffusion method (IZ). For MIC, MFC and IZ abbreviations: see Tab. 1. Literature abbreviations: [1] Kosanić et al. (2014c); [2] Ranković et al. (2007b); [3] Ranković et al. (2007a); [4] Ranković et al. (2010b); [5] Kosanić et al. (2010); [6] Ranković et al. (2011); [7] Ranković and Kosanić (2012); [8] Kosanić and Ranković (2011a);
[9] Ranković et al. (2012); [10] Kosanić et al. (2012b); [11] Ranković et al. (2009); [12] Kosanić and Ranković (2011b).

| **Lichen species** | **Extracting solvent** | **Results** | | | **Measurement method** | **Literature** |
| --- | --- | --- | --- | --- | --- | --- |
|  |  | **MIC [**mg·ml^-1^] | **MFC** | **IZ** [mm] (dose) |  |  |
| *Acarospora fuscata* | acetone | 10 | ‡ | ‡ | BMM | [1] |
| *Aspicilia cinerea* | acetone | † | ‡ | † | BTDM; DDM | [2] |
|  | methanol |  |  |  |  |  |
|  | water |  |  |  |  |  |
| *Lasallia pustulata* | acetone | 12.5 | ‡ | 12 (15 µl) | BTDM; DDM | [3] |
|  | methanol | 6.25 | ‡ | 19 (15 µl) |  |  |
|  | water | † | ‡ | † |  |  |
| *Lathagrium cristatum* | acetone | † | ‡ | † | BTDM; DDM | [2] |
|  | methanol |  |  |  |  |  |
|  | water |  |  |  |  |  |
| *Lecanora frustulosa* | acetone | † | ‡ | † | BTDM; DDM | [4], [5] |
|  | methanol | 12.5 | ‡ | 15 (15 µl) |  |  |
|  | water | † | ‡ | † |  |  |
| *Lecanora muralis* | acetone | † | ‡ | ‡ | BMM | [6] |
|  |  | † | ‡ | † | BTDM; DDM | [7] |
|  | methanol | 25 | ‡ | 13 (15 µl) | BTDM; DDM | [7] |
|  | water | † | ‡ | † | BTDM; DDM | [7] |
| *Physcia caesia* | acetone | † | ‡ | 20 (15 µl) | BTDM; DDM | [2] |
|  | methanol | 3.12 | ‡ | 23 (15 µl) |  |  |
|  | water | † | ‡ | † |  |  |
| *Tephromela atra* | acetone | 25 | ‡ | ‡ | BMM | [6] |
|  |  | 25 | ‡ | 12 (15 µl) | BTDM; DDM | [7], [8] |
|  | methanol | 3.12 | ‡ | 15 (15 µl) |  |  |
|  | water | † | ‡ | † |  |  |
| *Thalloidima candidum* | acetone | 25 | ‡ | ‡ | BMM | [9] |
| *Umbilicaria crustulosa* | acetone | 12.5 | ‡ | ‡ | BMM | [10] |
|  |  | 12.5 | ‡ | 14 (15 µl) | BTDM; DDM | [3] |
|  | methanol | 6.25 | ‡ | 18 (15 µl) |  |  |
|  | water | † | ‡ | † |  |  |
| *Umbilicaria cylindrica* | acetone | † | ‡ | † | BTDM; DDM | [3] |
|  |  | † | ‡ | ‡ | BMM | [10] |
|  | methanol | † | ‡ | † | BTDM; DDM | [3] |
|  | water | † | ‡ | † |  |  |
| *Umbilicaria polyphylla* | acetone | 12.5 | ‡ | 10 (15 µl) | BTDM; DDM | [11] |
|  |  | 12.5 | ‡ | ‡ | BMM | [10] |
|  |  | 12.5 | ‡ | ‡ |  | [12] |
|  | ethanol | 1.56 | ‡ | 30 (15 µl) | BTDM; DDM | [11] |
|  | methanol | 1.56 | ‡ | ‡ | BMM | [12] |
|  | water | † | ‡ | † | BTDM; DDM | [11] |
|  |  | † | ‡ | ‡ | BMM | [12] |
| *Xanthoparmelia xanthomelaena* | acetone | 2.5 | ‡ | ‡ | BMM | [1] |

† no effect, ‡ not investigated

Tab. S6. Effect of crude extracts from epigeic lichens on *Aspergillus niger*. Method of measurement abbreviations: BTDM = Broth tube dilution method (MIC); DDM = Disk diffusion method (IZ); MwR = Microdilution method with resazurin (MIC, MFC); AWDM = Agar well diffusion method (IZ). For MIC, MFC and IZ abbreviations: see Tab. 1. Literature abbreviations: [1] Bisht et al. (2014); [2] Ranković et al. (2010a); [3] Kosanić et al. (2018);
[4] Aslan et al. (2006); [5] Mitrović et al. (2011); [6] Ranković and Mišić (2007).

| **Lichen species** | **Extracting solvent** | **Results** | | | **Measurement method** | **Literature** |
| --- | --- | --- | --- | --- | --- | --- |
|  |  | **MIC [**mg·ml^-1^] | **MFC [**mg·ml^-1^] | **IZ** [mm] (dose) |  |  |
| *Cladonia* sp. | methanol | † | ‡ | † | BTDM; AWDM | [1] |
|  | water |  |  |  |  |  |
| *Cladonia digitata* | methanol | 30 | ‡ | 10 (10 µl) | BTDM; DDM | [2] |
| *Cladonia fimbriata* | acetone | 10 | ‡ | ‡ | MMwR | [3] |
|  | methanol | 15 | ‡ | 13 (10 µl) | BTDM; DDM | [2] |
| *Cladonia foliacea* | acetone | 10 | ‡ | ‡ | MMwR | [3] |
|  | methanol | ‡ | ‡ | † | DDM | [4] |
|  |  | 20 | 20 | † | MMwR | [5] |
|  |  | 10 | 10 | † |  |  |
| *Cladonia furcata* | acetone | 10 | ‡ | ‡ | MMwR | [3] |
| *Cladonia rangiferina* | acetone | 20 | ‡ | ‡ | MMwR | [3] |
|  | ethanol | 100 | 100 | 11 (20 µl) | BTDM; AWDM | [6] |
|  | ethyl acetate | 150 | 150 | 8 (20 µl) |  |  |
|  | water | >200 | † | † |  |  |
| *Cladonia subulata* | acetone | 10 | ‡ | ‡ | MMwR | [3] |
| *Gyalolechia fulgens* | methanol | † | ‡ | † | BTDM; DDM | [2] |
| *Peltigera* sp. | methanol | 5 × 10^-3^ | ‡ | 5.5 (10 µl) | BTDM; AWDM | [1] |
|  | water | 5 × 10^-3^ | ‡ | 2.87 (10 µl) |  |  |

† no effect, ‡ not investigated

Tab. S7. Effect of crude extracts from epilithic lichens on *Aspergillus niger*. Method of measurement abbreviations: BMM = Broth microdilution method (MIC); BTDM = Broth tube dilution method (MIC); DDM = Disk diffusion method (IZ); MMwR = Microdilution method with resazurin (MIC, MFC). For MIC, MFC and IZ abbreviations: see Tab. 1. Literature abbreviations: [1] Ranković et al. (2010c); [2] Manojlović et al. (2005);
[3] Aslan et al. (2006); [4] Ranković et al. (2010a); [5] Manojlović et al. (2012a); [6] Gulluce et al. (2006);
[7] Manojlović et al. (2002).

| **Lichen species** | **Extraction solvent** | **Results** | | | | **Measurement method** | **Literature** | **Literature** |
| --- | --- | --- | --- | --- | --- | --- | --- | --- |
|  |  | **MIC [**mg·ml^-1^] | **MFC** | **IZ** [mm] (dose) | **Other** |  |  |  |
| *Arctoparmelia centrifuga* | methanol | 15 | ‡ | 6 (10 µl) | ‡ | BMM; DDM | [5] | [1] |
| *Caloplaca cerina* | methanol | ‡ | ‡ | ‡ | IR: 45% | DDM | [2] | [2] |
| *Dermatocarpon miniatum* | methanol | ‡ | ‡ | † | ‡ | DDM | [7] | [3] |
| *Nephroma parile* | methanol | † | ‡ | † | ‡ | BMM; DDM | [5] | [1] |
| *Ochrolechia parella* | methanol | † | ‡ | † | ‡ | BTDM; DDM | [6] | [4] |
| *Ochrolechia tartarea* | methanol | 30 | ‡ | 7 (10 µl) | ‡ | BMM; DDM | [5] | [1] |
| *Umbilicaria cylindrica* | chloroform | 31.25 × 10^-3^ | ‡ | ‡ | ‡ | MMwR | [1] | [5] |
|  | methanol | 15.62 × 10^-3^ | ‡ | ‡ | ‡ |  |  |  |
| *Umbilicaria nylanderiana* | methanol | ‡ | ‡ | † | ‡ | DDM | [4] | [6] |
| *Variospora dolomiticola* | ethanol | 160 × 10^-3^ | ‡ | ‡ | ‡ | BTDM | [3] | [7] |
| *Xanthoparmelia pulla* | methanol | ‡ | ‡ | † | ‡ | DDM | [7] | [3] |

† no effect, ‡ not investigated, IR = inhibition rate

Tab. S8. Effect of lichen secondary metabolites on *Aspergillus nidulans*. Method of measurement abbreviations: BMM = Broth microdilution method (MIC). For MIC abbreviation: see Tab. 1. Literature abbreviations: [1] Hanuš et al. (2007); [2] Hanuš et al. (2008). Biochemical classes are given according to Elix (2014).

| **Biochemical class** | **Secondary metabolites** | **Extracting solvents** | **Results** | **Measurement method** | **Literature** |
| --- | --- | --- | --- | --- | --- |
|  |  |  | **MIC [**mg·ml^-1^] |  |  |
| monocyclic aromatic derivative | orsellinic acid | ethanol-water-hydrogen chloride | 6.7 × 10^-3^ | BMM | [1], [2] |
| orcinol depside | lecanoric acid | ethanol-water-hydrogen chloride | 10.3 × 10^-3^ | BMM | [1], [2] |
| β-orcinol depside | diffractaic acid | ethanol-water-hydrogen chloride | 9.1 × 10^-3^ | BMM | [1], [2] |
| β-orcinol depsidones | norstictic acid | ethanol-water-hydrogen chloride | 6.4 × 10^-3^ | BMM | [1], [2] |
|  | protocetraric acid |  | 11.6 × 10^-3^ |  |  |
| usnic acid derivative | usnic acid | ethanol-water-hydrogen chloride | 14.3 × 10^-3^ | BMM | [1], [2] |

Tab. S9. Effect of lichen secondary metabolites on *Aspergillus ochraceus*. Method of measurement abbreviations: BMM = Broth microdilution method (MIC). For MIC abbreviation: see Tab. 1. Literature abbreviations: [1] Hanuš et al. (2007); [2] Hanuš et al. (2008). Biochemical classes are given according to Elix (2014).

| **Biochemical class** | **Secondary metabolites** | **Extracting solvents** | **Results** | **Measurement method** | **Literature** |
| --- | --- | --- | --- | --- | --- |
|  |  |  | **MIC [**mg·ml^-1^] |  |  |
| monocyclic aromatic derivative | orsellinic acid | ethanol-water-hydrogen chloride | 14.5 × 10^-3^ | BMM | [1], [2] |
| orcinol depside | lecanoric acid | ethanol-water-hydrogen chloride | 18.2 × 10^-3^ | BMM | [1], [2] |
| β-orcinol depside | diffractaic acid | ethanol-water-hydrogen chloride | 5.2 × 10^-3^ | BMM | [1], [2] |
| β-orcinol depsidones | norstictic acid | ethanol-water-hydrogen chloride | 13.8 × 10^-3^ | BMM | [1], [2] |
|  | protocetraric acid |  | 7.4 × 10^-3^ |  |  |
| usnic acid derivative | usnic acid | ethanol-water-hydrogen chloride | 11.3 × 10^-3^ | BMM | [1], [2] |

Tab. S10. Effect of lichen secondary metabolites on *Aspergillus parasiticus*. Method of measurement abbreviations: BMM = Broth microdilution method (MIC); MMwR = Microdilution method with resazurin (MIC, MFC). For MIC abbreviation: see Tab. 1. Literature abbreviations: [1] Sarıözlü et al. (2016); [2] Hanuš et al. (2007); [3] Hanuš et al. (2008); [4] Cankılıç et al. (2017). Biochemical classes are given according to Elix (2014).

| **Biochemical class** | **Secondary metabolites** | **Extracting solvents** | **Results** | **Measurement method** | **Literature** |
| --- | --- | --- | --- | --- | --- |
|  |  |  | **MIC [**mg·ml^-1^] |  |  |
| benzyl esters | barbatolic acid | methanol, chloroform and acetone | 400 × 10^-3^ | MMwR | [1] |
| monocyclic aromatic derivatives | orsellinic acid | ethanol-water-hydrogen chloride | 7.8 × 10^-3^ | BMM | [2], [3] |
| orcinol depsides | lecanoric acid | ethanol-water-hydrogen chloride | 11.9 × 10^-3^ | BMM | [2], [3] |
| β-orcinol depsides | diffractaic acid | ethanol-water-hydrogen chloride | 5.9 × 10^-3^ | BMM | [2], [3] |
|  | thamnolic acid | methanol, chloroform and acetone | † | BMM | [4] |
| β-orcinol depsidones | norstictic acid | ethanol-water-hydrogen chloride | 12.4 × 10^-3^ | BMM | [2], [3] |
|  | protocetraric acid | ethanol-water-hydrogen chloride | 9.3 × 10^-3^ | BMM | [2], [3] |
| usnic acid derivatives | usnic acid | ethanol-water-hydrogen chloride | 6.4 × 10^-3^ | BMM | [2], [3] |

† no effect

Tab. S11. Lichen secondary metabolites of species with significant inhibitory potential against *Aspergillus* species. Abbreviations: trace = trace concentration of the secondary metabolite in the obtained extract; MC = secondary metabolites with the highest concentration in the obtained extract. Literature abbreviations: [1] Nash III et al. (2002); [2] Mitrović et al. (2011); [3] Smith et al. (2009); [4] Mitrović et al. (2014); [5] Aslan et al. (2006);
[6] Manojlović et al. (2012a); [7] Manojlović et al. (2002).

| ***Aspergillus* species** | **Lichen species** | **Extracting solvent** | **Secondary compounds** | **Literature** |
| --- | --- | --- | --- | --- |
| *Aspergillus candidus* | *Hypotrachyna cirrhata* | water | atranorin  chloroatranorin  consalazinic acid  galbinic acid  protocetraric acid  salazinic acid | [1] |
| *Aspergillus flavus* | *Cladonia foliacea* | methanol | atranorin  fumarprotocetraric acid  usnic acid | [2] |
|  | *Hypotrachyna cirrhata* | water | see: as above | [1] |
|  | *Leucodermia leucomelos* | water | salazinic acid  zeorin | [3] |
|  | *Platismatia glauca* | methanol | 5,7-dihydroxy-6-methylphtalide  dimethyl caperate (MC)  isoadiantone  methyl chlorohaematommate  methyl haematommate  methyl oleate (trace)  methyl orsellinate  methyl palmitate  methyl β-orcinolcarboxylate  olivetol | [4] |
|  | *Pseudevernia furfuracea* | acetone | 5-(2-oxoheptyl)-resorcinol  atranol  chloroatranol  ethyl haematommate (trace)  methyl 2,4-dihydroxy-3,5,6-trimethylbenzoate  methyl haematommate  methyl orsellinate  methyl β-orcinolcarboxylate (MC)  olivetol  olivetonide | [4] |
|  |  | methanol | 5-(2-oxoheptyl)-resorcinol  atranol (trace)  chloroatranol (trace)  methyl 2,4-dihydroxy-3,5,6-trimethylbenzoate (trace)  methyl chlorohaematommate  methyl haematommate  methyl linoleate  methyl orsellinate  methyl palmitate (trace)  methyl β-orcinolcarboxylate (MC)  olivetol  olivetonide |  |
| *Aspergillus fumigatus* | *Cladonia foliacea* | methanol | see: as above | [2] |
|  | *Nephroma arcticum* | water | nephroarctin  phenarctin  ± usnic acid  zeorin | [3] |
|  | *Parmelia sulcata* | methanol | arabinitol  atranol  atraric acid  divaricatic acid  ergosterol  lichesterol  linoleic acid  linolenic acid  methyl haematommate  nonacosane  oleic acid  olivetol  palmitic acid  salazinic acid  stearic acid  α-tocopherol  β-sitosterol | [2] |
| *Aspergillus niger* | *Evernia prunastri* | methanol | arabinitol  atranol  atraric acid  ergosterol  evernic acid  lichesterol  linoleic acid  methyl haematommate  methyl orsellinate  oleic acid  orcinol  orcinol monomethylether  orsellinic acid  palmitic acid  sparassol  stearic acid  usnic acid | [5] |
|  | *Hypogymnia physodes* | methanol | 2'-O-methylphysodic acid  3-hydroxyphysodic acid  atranol  atraric acid  chloroatranol  ergosterol  isophysodic acid  lichesterol  linoleic acid  methyl haematommate  oleic acid  olivetol  olivetonic acid  olivetonide  orcinol  palmitic acid  physodalic acid  physodic acid  stearic acid  α-tocopherol | [2] |
|  | *Umbilicaria cylindrica* | methanol  chloroform | atranorin  ethyl haematommate  methyl β-orcinolcarboxylate  norstictic acid  salazinic acid  usnic acid | [6] |
|  | *Variospora dolomiticola* | ethanol | emodin  fallacinal  fallacinol  parietin  parietinic acid | [7] |
| *Aspergillus parasiticus* | *Hypotrachyna cirrhata* | water | see: as above | [1] |
|  | *Leucodermia leucomelos* | water | see: as above | [3] |
| *Aspergillus restrictus* | *Evernia prunastri* | methanol | see: as above | [5] |
|  | *Hypogymnia physodes* | methanol | see: as above | [2] |
|  | *Parmelia sulcata* | methanol | see: as above | [2] |
| *Aspergillus ustus* | *Hypotrachyna cirrhata* | water | see: as above | [1] |

Tab. S12A-B. Activity of antifungal non-lichen substances against *Aspergillus* fungi follow: [1] Schmeda-Hirschmann et al. (2008); [2] Babiah et al. (2014a);
[3] Hanuš et al. (2007); [4] Tiwari et al. (2011a); [5] Anjali et al. (2015b); [6] Esimone and Adikwu (1999); [7] Ranković et al. (2009); [8] Stojanović et al. (2013); [8] Ranković et al. (2010b); [9] Ranković et al. (2007b); [10] Thadhani et al. (2012); [11] Hoda and Vijayaraghavan (2015); [12] Ranković et al. (2010a);
[13] Ranković et al. (2010c); [14] Aslan et al. (2006); [15] Kosanić et al. (2012a); [16] Kosanić et al. (2012b); [17] Ranković et al. (2011); [18] Mitrović et al. (2011); [19] Tiwari et al. (2011b); [20] Kosanić et al. (2014c); [21] Ranković et al. (2012); [22] Manojlović et al. (2012a); [23] Manojlović et al. (2012b); [24]Ranković et al. (2014b); [25] Kosanić et al. (2014b); [26] Anjali et al. (2015a); [27] Ranković et al. (2007a); [28] Kosanić et al. (2014a); [29] Grujičić et al. (2014); [30] Kosanić et al. (2013a); [31] Babiah et al. (2014b); [32] Kosanić et al. (2013b); [33] Kirmizigül et al. (2003); [34] Kosanić et al. (2016); [35] Mitrović et al. (2014); [36] Sasidharan et al. (2014); [37] Gulluce et al. (2006); [38] Ranković et al. (2014a); [39] Praveen Kumar et al. (2010); [40] Ranković et al. (2008); [41] Ranković and Mišić (2008); [42] Ranković and Kosanić (2012); [43] Kosanić et al. (2010); [44] Cankılıç et al. (2017); [45] Ristić et al. (2016); [46] Sarıözlü et al. (2016); [47] Hanuš et al. (2008); [49] Kosanić et al. (2018); [50] Maulidiyah et al. (2018); [51] Akinpelu et al. (2015); [52] Day et al. (2009); [53] Mello
et al. (2017); [54] Baddley et al. (2009); [55] Negri et al. (2014); [56] Sarrafha et al. (2018); [57] Al-Wathiqi et al. (2013); [58] Denardi et al. (2018); [59] Li et al. (2020).

* = not given; † = MIC_90_/MFC_90_. All values are given in mg·ml^-1^ for MIC/MFC or in mm for IZ. In some cases, units have been converted to standardize the results.

| Activity of antifungal non-lichen substances (part A) | *A. flavus* | | | *A. fumigatus* | | | *A. niger* | | |
| --- | --- | --- | --- | --- | --- | --- | --- | --- | --- |
|  | MIC | MFC | IZ | MIC | MFC | IZ | MIC | MFC | IZ |
| Amphotericin B | 0.064-4 × 10^-3^ [57];  0.5 × 10^-3^ [1];  0.5-1 × 10^-3^ [54];  0.5-8 × 10^-3^ [58];  2-8 × 10^-3^ [59] 22 × 10^-3^ [10]; 4 × 10^-3^ [36]; 15.62 × 10^-3^ [14], [37] | * | 7-16 [57];  23 [36] | 0.06-1 × 10^-3^ [58];  0.125-2 × 10^-3^ [54];  0.5 × 10^-3^[1]; 1-4 × 10^-3^ [59] | * | * | 0.125-0.25 × 10^-3^ [54];  0.5 × 10^-3^ [1];  0.5-4 × 10^-3^ [59]; 15.62 × 10^-3^ [14], [37] | * | * |
| Amoxycyllin | >4^†^ [52] | * | * | * | * | * | * | * | * |
| Anidulafungin | 0.002-0.016 × 10^-3^ [57]; 0.002-0.125 × 10^-3^ [58]; ≤0.015 × 10^-3^ [59] | * | * | 0.001-0.125 × 10^-3^ [58]; ≤0.015-0.06 × 10^-3^ [59] | * | * | ≤0.015 × 10^-3^ [59] | * | * |
| Benzalkonium chloride | 16 × 10^-3 †^ [52] | 32 × 10^-3 †^ [52] | * | * | * | * | * | * | * |
| Caspofungin | 0.002-0.125 × 10^-3^ [57];  ≤0.008-0.03 × 10^-3^ [59] 0.008-0.250 × 10^-3^ [58] | * | * | ≤0.008-0.03 × 10^-3^ [59]  0.03-1 × 10^-3^ [58]; | * | * | ≤0.008-0.03 × 10^-3^ [59] | * | * |
| Cefazolin | >4^†^ [52] | * | * | * | * | * | * | * | * |
| Chloramphenicol | 4^†^ [52] | >4^†^ [52] | * | * | * | * | * | * | * |
| Clotrimazole | 18.1 x10^-3^ [3], [47] | * | * | * | * | * | 19.3 x10^-3^[3], [47] | * | 18 [6] |
| Fluconazole | 16-256 × 10^-3^ [56]  1 [18], [35] | 1 [18], [35] | * | 5 × 10^-1^ [18]; 128-256 × 10^-3^ [56] | 1 [18] | 18.2 [11]; 26 [39] | 6.25 × 10^-2^ [18];  5 × 10^-1^ [18] | 6.25 × 10^-2^ [18];  1 [18] | 16.4 [11]; 24 [39] |
| Itraconazole | ≤0.03-0.25 × 10^-3^ [53];  0.03-0.25 × 10^-3^ [59] 0.06-0.25 × 10^-3^ [54]; 0.5-8 × 10^-3^ [58] | * | * | 0.12-2 × 10^-3^ [53];  0.125-4 × 10-^3^ [54];  ≤0.015-16 × 10^-3^ [59] 1-16 × 10^-3^ [58]; >8 × 10^-3^ [53]; | * | * | ≤0.015-4 × 10^-3^ [59];  0.12-0.5 × 10^-3^ [53]; 0.25-1 × 10^-3^ [54] | * | * |
| Ketoconazole | 0.125 × 10^-3^ [1];  3.75 × 10^-3^[12]; 3.9 × 10^-3^ [7], [8], [9], [20], [21], [23], [24], [25], [27], [28], [30], [32], [40], [41], [42], [43];  7.81 × 10^-3^ [29], [34], [38]; 39.06 × 10^-3^ [46];  312 × 10^-3^[45], [49] | 3.9 × 10^-3^ [15], [16], [17];  7.5 × 10^-3^[13] | c. 8-23 [19];  9.33 [26];  14 [5];  19 [13];  20 [4];  22 [2], [31];  24 [50]  27  [7], [8], [9], [12], [27], [42], [43];  28 [46]; 32 [33]; 35 [33]; | 0.25 × 10^-3^ [1]; 3.9 × 10^-3^ [7], [8], [9], [15], [16], [17], [20], [21], [23], [24], [25], [27], [30], [32], [40], [41], [42], [43]; 78.12 × 10^-3^ [44], [46] | * | 10 [4];  c. 15-22 [19]; 28 [33]; 34 [7], [8], [9], [42], [43]; 35 [44], [46] | 0.5 × 10^-3^ [1];  0.97 × 10^-3^ [22]; 3.75 × 10^-3^ [12]; 7.5 × 10^-3^ [13]; 39.06 × 10^-3^ [46]; 78 × 10^-3^ [45], [49] | * | 9 [5];  12.66 [26];  21 [13];  24.3 [2], [31];  22.6 [4];  34 [12], [46] |
| Micafungin | 0.002-0.008 × 10^-3^ [57]; 0.004-0.25 × 10^-3^ [58]; ≤0.008-0.03 × 10^-3^ [59] | * | * | 0.001-1 × 10^-3^ [58]; ≤0.008-0.03 × 10^-3^ [59] | * | * | ≤0.008-0.03 × 10^-3^ [59] | * | * |
| Moxifloxacin | >4^†^ [52] | * | * | * | * | * | * | * | * |
| Nystatin | * | * | * | * | * | 25 [51] | * | * | 18.3 [8]; 24 [51] |
| Posaconazole | ≤0.008-2 × 10^-3^ [59];  0.016-0.5 × 10^-3^ [57];  0.03-0.25 × 10^-3^ [58];  0.06-0.12 × 10^-3^ [53]; 0.06-0.125 × 10^-3^ [54] | * | * | ≤0.008-0.25 × 10^-3^ [59];  0.03-0.5 × 10^-3^ [58];  0.03-1 × 10^-3^ [54];  0.06-0.12 × 10^-3^ [53]; 0.5-1 × 10^-3^ [53] | * | * | ≤0.008-0.25 × 10^-3^ [59];  ≤0.03-0.25 × 10^-3^ [53]; 0.06-0.5 × 10^-3^ [54] | * | * |
| Ravuconazole | 0.25-0.5 × 10^-3^ [54] | * | * | 0.25-1 × 10^-3^ [54] | * | * | * | * | * |
| Tobramycin | >4^†^ [52] | * | * | * | * | * | * | * | * |
| Voriconazole | 0.012-8 × 10^-3^ [59];  0.064-0.25 × 10^-3^ [57];  0.12-1 × 10^-3^ [53]; 0.125-1 × 10^-3^ [54]; 0.5-2 × 10^-3^ [58]; | * | 24-34 [57] | 0.06-8 × 10^-3^ [59]  0.12->8 × 10^-3^ [53]; 0.125-8 × 10^-3^ [54]; 0.25-4 × 10^-3^ [58] | * | * | 0.06-2 × 10^-3^ [59];  0.25-0.5 × 10^-3^ [53]; 0.5-1 × 10^-3^ [54] | * | * |

| Activity of antifungal non-lichen  substances (part B) | *A. candidus* | | *A. nidulans* | | *A. ochraceus* | | *A. parasiticus* | | *A. restrictus* | | | *A. stellatus* | | *A. ustus* | |
| --- | --- | --- | --- | --- | --- | --- | --- | --- | --- | --- | --- | --- | --- | --- | --- |
|  | MIC | IZ | MIC | IZ | MIC | IZ | MIC | IZ | MIC | MFC | IZ | MIC | IZ | MIC | IZ |
| Amphotericin B | * | * | 2-4 × 10^-3^ [59] | * | * | * | * | * | * | * | * | 15.62 × 10^-3^ [14], [37] | * | 2 × 10^-3^ [59] | * |
| Anidulafungin | * | * | ≤0.015 × 10^-3^ [59] | * | * | * | * | * | * | * | * | * | * | ≤0.015 × 10^-3^ [59] | * |
| Caspofungin | * | * | ≤0.008-0.03 × 10^-3^  [59] | * | * | * | * | * | * | * | * | * | * | 0.03 × 10^-3^ [59] | * |
| Clotrimazole | * | * | 15.6 × 10^-3^ [3]; 15.6 × 10^-3^ [47] | * | 20.4 × 10^-3^ [3], [47] | * | 16.2 × 10^-3^ [3], [47] | * | * | * | * | * | * | * | * |
| Fluconazole | * | * | * | * | * | * | * | * | 5 × 10^-1^  [18] | 2  [18] | * | * | * | * | * |
| Itraconazole | * | * | ≤0.015-4 × 10^-3^ [59];  0.12-0.25 × 10^-3^ [53] | * | 4 × 10^-3^  [55] | * | * | * | * | * | * | * | * | 0.5 × 10^-3^ [59] | * |
| Ketoconazole | * | * | * | * | * | * | 78.12 × 10^-3^  [46] | 35  [46] | * | * | * | * | * | * | * |
| Micafungin | * | * | ≤0.008-0.03 × 10^-3^  [59] | * | * | * | * | * | * | * | * | * | * | 0.06 × 10^-3^ [59] | * |
| Nystatin | * | * | * | * | * | * | * | * | * | * | * | * | * | * | * |
| Posaconazole | * | * | 0.015-0.06 × 10^-3^ [59];  0.06-0.12 × 10^-3^ [53] | * | 0.5 × 10^-3^  [55] | * | * | * | * | * | * | * | * | 1 × 10^-3^ [59] | * |
| Voriconazole | * | * | 0.03-2 × 10^-3^ [59];  0.12-0.12 × 10^-3^ [53] | * | 1 × 10^-3^  [55] | * | * | * | * | * | * | * | * | 8 × 10^-3^ [59] | * |
